# Efficacy and safety of esaxerenone with and without sodium–glucose cotransporter-2 inhibitor use in hypertensive patients with type 2 diabetes mellitus: a pooled analysis of five clinical studies

**DOI:** 10.1038/s41440-025-02347-0

**Published:** 2025-09-01

**Authors:** Hirohiko Motoki, Koichiro Kuwahara, Haruhito A. Uchida, Jun Wada, Kazuomi Kario, Tomohiro Katsuya, Tatsuo Shimosawa, Kenichi Tsujita, Shoko Suzuki, Tomohiro Suedomi, Takashi Taguchi

**Affiliations:** 1 Department of Cardiovascular Medicine, Shinshu University School of Medicine, Nagano, Japan; 2 Department of Nephrology, Rheumatology, Endocrinology and Metabolism, Okayama University Faculty of Medicine, Dentistry and Pharmaceutical Sciences, Okayama, Japan; 3 Division of Cardiovascular Medicine, Department of Medicine, Jichi Medical University School of Medicine, Tochigi, Japan; 4 Katsuya Clinic, Hyogo, Japan; 5 Department of Clinical Laboratory, School of Medicine, International University of Health and Welfare, Chiba, Japan; 6 Department of Cardiovascular Medicine, Graduate School of Medical Sciences, Kumamoto University, Kumamoto, Japan; 7 Data Intelligence Department, Daiichi Sankyo Co. Ltd., Tokyo, Japan; 8 Primary Medical Science Department, Daiichi Sankyo Co. Ltd., Tokyo, Japan

**Keywords:** Esaxerenone, Hypertension, Morning home blood pressure, Sodium–glucose cotransporter-2 inhibitor, Type 2 diabetes mellitus

## Abstract

This pooled subanalysis of five multicenter, prospective, open-label, single-arm studies on esaxerenone aimed to evaluate the efficacy, organ-protective effects, and safety of esaxerenone in hypertensive patients with type 2 diabetes mellitus (T2DM), with and without concomitant sodium–glucose cotransporter-2 inhibitor (SGLT2i) therapy. In total, 283 and 279 patients were included in the safety (with SGLT2i, 148; without, 135) and full analysis sets (with SGLT2i; 145; without, 134), respectively. Significant changes in morning home systolic/diastolic blood pressure (SBP/DBP) from baseline to Week 12 were shown in the overall population (mean change: −11.9/−5.2 mmHg, both *P* < 0.001) and both SGLT2i and non-SGLT2i subgroups (−11.3/−4.8 and −12.5/−5.7 mmHg, respectively, all *P* < 0.001). Similar findings were observed in bedtime home and office SBP/DBP. The proportions of patients who achieved target home SBP/DBP < 135/85 mmHg were 71.2% (overall population) and 70.5% and 71.9% in the SGLT2i and non-SGLT2i subgroups, respectively. The urine albumin-to-creatinine ratio significantly improved from baseline to Week 12 in the overall population and SGLT2i subgroups (percentage change in geometric mean from baseline: −42.8%, −43.0%, and −42.6%, respectively, all *P* < 0.001). N-terminal pro-B-type natriuretic peptide levels improved in all groups. The incidence of serum potassium ≥5.5 mEq/L was 2.0% vs 5.2% in the SGLT2i vs non-SGLT2i subgroups. Esaxerenone demonstrated significant BP-lowering effects, and improved renal and cardiovascular parameters, regardless of SGLT2i use. Safety was consistent across groups, with the numerically lower incidence of serum potassium ≥5.5 mEq/L in the SGLT2i subgroup suggesting a potential mitigating effect of SGLT2is on the risk of hyperkalemia.

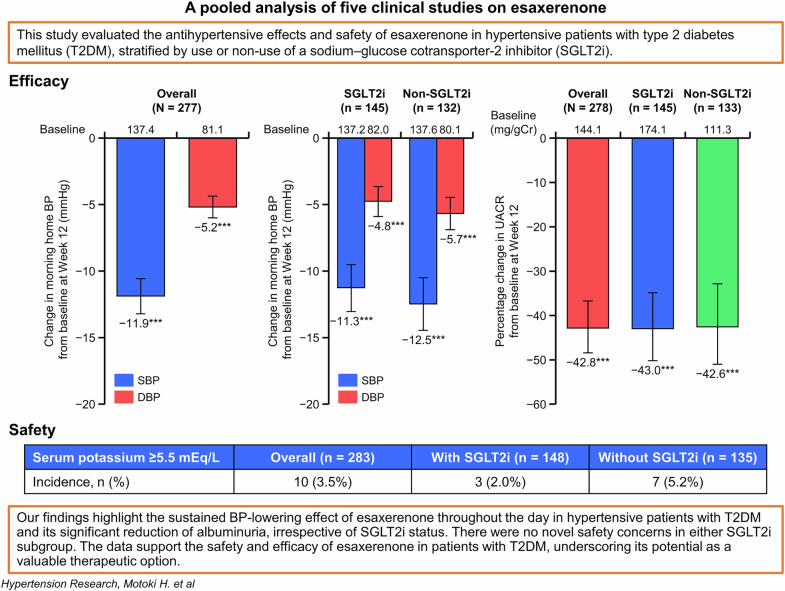

## Introduction

Hypertension is a major contributor to cardiovascular and cerebrovascular events, and cerebrovascular morbidity and mortality, as well as a key driver in the progression of diabetic nephropathy [1–9]. The adverse outcomes associated with these conditions are closely linked to elevated blood pressure (BP) levels, necessitating effective management strategies to mitigate these risks. In patients with type 2 diabetes mellitus (T2DM), hypertension is a major cardiovascular risk factor [10], and hypertension and T2DM often co-exist. Such co-existence is expected to become more common as the population ages [11, 12].

The 2019 Japanese Society of Hypertension (JSH) guidelines recommend mineralocorticoid receptor blockers (MRBs) as fourth-line treatment for hypertension, including in patients with both hypertension and T2DM [13]. MRBs have not only antihypertensive effects but also cardioprotective and renoprotective effects [14–17]. The MRB finerenone has shown renoprotective effects in patients with chronic kidney disease (CKD) and T2DM [18], but is not currently approved for the treatment of hypertension. While MRBs, such as spironolactone and eplerenone, are effective in reducing BP, their use is often complicated by adverse effects, including decline in kidney function. Although non-steroidal MRBs also cause decline in kidney function, albeit to a lesser extent, spironolactone and eplerenone are contraindicated in patients with reduced kidney function (estimated glomerular filtration rate [eGFR] <60 mL/min/1.73 m^2^ according to the Japanese package insert and have limited applicability in hypertensive patients with T2DM, especially in the Japanese clinical setting.

Esaxerenone, a next-generation non-steroidal MRB, has a higher selectivity and potency, a longer half-life, and more favorable bioavailability compared with other MRBs [19, 20]. Esaxerenone has been shown to elicit favorable antihypertensive effects in hypertensive patients with various characteristics [21–25], including patients with T2DM [26–29]. In addition to its antihypertensive effects, esaxerenone has also demonstrated renoprotective effects, such as the reduction and remission of albuminuria [26–29]. Despite these advantages, MRB-induced hyperkalemia is an important clinical concern, especially in patients with kidney impairment and diabetes. However, this issue can be managed by dose adjustment based on regular monitoring of serum potassium levels [21].

Sodium–glucose cotransporter-2 inhibitors (SGLT2is) have demonstrated cardioprotective and renoprotective effects [30, 31] and elicit slight reductions in BP through improved glycemic control [32, 33]. They currently play a central role in the treatment of T2DM. Several studies have reported that combining SGLT2is with MRBs can reduce the risk of hyperkalemia in patients with T2DM [34–36]. A subgroup analysis of phase 3 clinical trials on esaxerenone has also shown that although serum potassium levels increase, the risk of hyperkalemia is mitigated by concomitant SGLT2i use without compromising the antihypertensive effects or urine albumin-to-creatinine ratio (UACR)-lowering effects of MRBs [37]. Similar findings were obtained in the EAGLE-DH study, a multicenter, open-label, prospective, interventional study of the efficacy and safety of esaxerenone in hypertensive patients with T2DM receiving SGLT2is [26]. However, most evidence regarding the combined use of esaxerenone and SGLT2is has come from a limited number of clinical studies and lacks diversity in patient backgrounds reflective of real-world clinical practice. Consequently, further investigation is warranted to confirm efficacy and safety in a more diverse, real-world T2DM population, irrespective of SGLT2i use.

The purpose of the present study was to evaluate the efficacy, organ-protective effects, and safety of esaxerenone in hypertensive patients with T2DM, with or without concomitant SGLT2i therapy, by pooling data from five clinical studies.

## Methods

### Study design

This study was a pooled subanalysis [38] of the following five clinical studies on esaxerenone: EX-DKD [22], EARLY-NH [23], ESES-LVH [24], ENaK [25], and EAGLE-DH [26]. These studies were multicenter, prospective, open-label, single-arm studies. The target populations of each study are described in Supplementary Table 1. In these studies, patients received esaxerenone in combination with basal antihypertensive medications including angiotensin receptor blockers (ARBs), calcium channel blockers (CCBs), or renin–angiotensin system (RAS) inhibitors. Esaxerenone dosing was adjusted at the treating physician’s discretion according to the patient’s condition (antihypertensive effect and serum potassium level) and the package insert.

This subanalysis included hypertensive patients with T2DM from the five clinical studies on esaxerenone [22–26]. Patients enrolled in this subanalysis were divided into two subgroups based on use/non-use of an SGLT2i. The respective eligibility criteria and methods for measuring BP and biomarkers have been previously reported in detail [22–26]. Of note, while the EAGLE-DH and ESES-LVH studies had 24-week treatment periods, this subanalysis used data up to Week 12 from all five studies.

The present study received ethical approval from the ethical review committee of the Kitamachi Clinic (Tokyo, Japan) and was conducted in accordance with the ethical principles outlined in the Declaration of Helsinki and local laws and regulations. Informed consent was waived because this study involved the secondary use of data from previous studies, and the data were fully de-identified prior to access. The five studies included in this pooled analysis were registered at the Japan Registry of Clinical Trials (jRCT) under the following identifiers: jRCTs061190027 (EX-DKD), jRCTs031200364 (EARLY-NH), jRCTs071190043 (ESES-LVH), jRCTs031210273 (ENaK), and jRCTs031200273 (EAGLE-DH). The present study is registered at the University hospital Medical Information Network Clinical Trials Registry (UMIN) under the identifier UMIN000051525.

### Study endpoints

The following efficacy endpoints were evaluated in the present subanalysis: time-course change and change from baseline to Week 12 in morning home, bedtime home, and office systolic/diastolic BP (SBP/DBP); achievement rate of target BP levels; and change and percentage change from baseline to Week 12 in UACR and N-terminal pro-B-type natriuretic peptide (NT-proBNP).

The safety endpoints included treatment-emergent adverse events (TEAEs), adverse drug reactions (ADRs), change from baseline and time-course change in creatinine-based eGFR (eGFR_creat_) and serum potassium levels, and proportion of patients with a serum potassium level ≥5.5 mEq/L. ADRs were defined as any adverse event for which a causal relationship with the study drug could not be ruled out.

The exploratory endpoints were UACR improvement rate, proportion of patients with a ≥30% reduction in UACR from baseline, and UACR remission rate. UACR remission was defined as transition to UACR < 30 mg/gCr (UACR A1) from UACR 30– < 300 mg/gCr (UACR A2) or UACR ≥ 300 mg/gCr (UACR A3), along with a ≥30% reduction in UACR from baseline.

### Statistical analysis

The sample size was not prespecified because this was an additional subanalysis. The efficacy endpoints were primarily evaluated in the full analysis set (FAS) of each study and evaluated in the per-protocol set (PPS) as a sensitivity analysis. The safety endpoints were evaluated in the safety analysis set of each study.

Descriptive statistics were used to summarize patient background characteristics, including mean ± standard deviation (SD) for continuous data and *n* (%) for categorical data. For the differences in BP measurements between baseline and Week 12, point estimates and 95% confidence intervals (CIs) were calculated and compared using paired t-tests. Similar significance tests were applied to the change and percentage change from baseline in UACR and NT-proBNP. The 95% CIs of the achievement rate of target BP levels were calculated using the Clopper–Pearson method. Missing values at Week 12 were not imputed in this study. TEAEs and ADRs were coded by System Organ Class and Preferred Term according to the Medical Dictionary for Regulatory Activities, version 27.0. The significance level was set at 5% (two-sided). All statistical analyses were performed using SAS version 9.4 (SAS Institute Inc., Cary, NC, USA).

## Results

### Patients

In the primary analysis [38], 492, 479, and 445 patients were included in the safety analysis set, FAS, and PPS of the five esaxerenone studies, respectively. Among them, the present subanalysis included 283 patients (with SGLT2i, *n *= 148; without, *n *= 135) in the safety analysis set, 279 patients (with SGLT2i, *n *= 145; without, *n *= 134) in the FAS, and 253 patients (with SGLT2i, *n *= 132; without, *n *= 121) in the PPS, respectively.

The background characteristics of patients in the FAS and PPS are shown in Table 1 and Supplementary Table 2, respectively. The SGLT2i subgroup included a greater proportion of male than female patients (69.0% vs 31.0%, respectively), while the non-SGLT2i subgroup had a nearly equal male-to-female distribution (49.3% vs 50.7%, respectively). Mean ± SD age was 66.5 ± 9.9 and 69.4 ± 10.4 years and body mass index was 27.4 ± 4.2 and 25.3 ± 3.8 kg/m^2^ in the SGLT2i and non-SGLT2i subgroups, respectively. Mean morning home SBP/DBP was similar in both subgroups (137.2/82.0 vs 137.6/80.1 mmHg, respectively). Mean ± SD serum potassium levels were similar in both subgroups (4.3 ± 0.4 vs 4.2 ± 0.4 mEq/L, respectively). Mean ± SD eGFR_creat_ was higher in the SGLT2i subgroup than the non-SGLT2i subgroup (65.1 ± 19.8 vs 58.0 ± 15.4 mL/min/1.73 m^2^, respectively). In the overall population, the distribution of patients using basal antihypertensive drugs was 40.5% for RAS inhibitors, 17.6% for CCBs, and 41.9% for both drugs; the distribution of basal antihypertensive drugs was similar in both subgroups. In the SGLT2i subgroup, the final doses of esaxerenone were 1.25, 2.5, and 5 mg in 26.2%, 49.0% and 24.8% of patients, respectively, vs 43.3%, 38.8%, and 17.9% of patients in the non-SGLT2i subgroup.

**Table 1:** Patient characteristics (full analysis set)

Characteristic	Overall *N *= 279	SGLT2i *n *= 145	Non-SGLT2i *n *= 134
Sex, male	166 (59.5)	100 (69.0)	66 (49.3)
Age, years	67.9 ± 10.2	66.5 ± 9.9	69.4 ± 10.4
≥65	193 (69.2)	90 (62.1)	103 (76.9)
BMI, kg/m^2^	26.4 ± 4.1 *n *= 278	27.4 ± 4.2 *n *= 144	25.3 ± 3.8 *n *= 134
≥25	168 (60.2)	100 (69.0)	68 (50.7)
Initial esaxerenone dose, mg/day	1.7 ± 0.6	1.8 ± 0.6	1.6 ± 0.6
1.25	180 (64.5)	85 (58.6)	95 (70.9)
2.5	99 (35.5)	60 (41.4)	39 (29.1)
Final esaxerenone dose, mg/day	2.6 ± 1.4	2.8 ± 1.4	2.4 ± 1.3
1.25	96 (34.4)	38 (26.2)	58 (43.3)
2.5	123 (44.1)	71 (49.0)	52 (38.8)
5	60 (21.5)	36 (24.8)	24 (17.9)
Dose escalation from the first dose of esaxerenone to 12 weeks	121 (43.4)	66 (45.5)	55 (41.0)
Current smoker	45 (16.1)	29 (20.0)	16 (11.9)
Alcohol use	103 (36.9)	62 (42.8)	41 (30.6)
Complications	277 (99.3)	144 (99.3)	133 (99.3)
Dyslipidemia	223 (79.9)	116 (80.0)	107 (79.9)
Hyperuricemia	78 (28.0)	39 (26.9)	39 (29.1)
Heart failure	31 (11.1)	20 (13.8)	11 (8.2)
Disease duration of hypertension, years	10.7 ± 8.3 *n *= 195	10.6 ± 8.1 *n *= 105	10.8 ± 8.6 *n *= 90
Basal antihypertensive agents			
RAS inhibitor	113 (40.5)	50 (34.5)	63 (47.0)
CCB	49 (17.6)	27 (18.6)	22 (16.4)
Both RAS inhibitor/CCB	117 (41.9)	68 (46.9)	49 (36.6)
Diabetes drug class			
SGLT2i	145 (52.0)	145 (100.0)	0 (0.0)
Biguanide	118 (42.3)	55 (37.9)	63 (47.0)
Thiazolidinedione	13 (4.7)	5 (3.4)	8 (6.0)
Sulfonylurea	41 (14.7)	18 (12.4)	23 (17.2)
Glinide	23 (8.2)	9 (6.2)	14 (10.4)
DPP-4 inhibitor	162 (58.1)	85 (58.6)	77 (57.5)
Alpha-glucosidase inhibitor	25 (9.0)	5 (3.4)	20 (14.9)
Insulin	24 (8.6)	8 (5.5)	16 (11.9)
GLP1 agonist	14 (5.0)	6 (4.1)	8 (6.0)
Number of oral diabetes medications			
1	72 (25.8)	35 (24.1)	37 (27.6)
2	85 (30.5)	42 (29.0)	43 (32.1)
≥3	99 (35.5)	68 (46.9)	31 (23.1)
None	23 (8.2)	0 (0.0)	23 (17.2)
Morning home SBP, mmHg	137.4 ± 11.7 *n *= 277	137.2 ± 11.2 *n *= 145	137.6 ± 12.2 *n *= 132
Morning home DBP, mmHg	81.1 ± 10.3 *n *= 277	82.0 ± 9.4 *n *= 145	80.1 ± 11.3 *n *= 132
Bedtime home SBP, mmHg	132.2 ± 13.3 *n *= 274	132.5 ± 12.6 *n *= 142	132.0 ± 14.1 *n *= 132
Bedtime home DBP, mmHg	76.5 ± 11.2 *n *= 274	77.8 ± 10.8 *n *= 142	75.1 ± 11.4 *n *= 132
Office SBP, mmHg	140.6 ± 14.0	138.5 ± 14.8	142.8 ± 12.7
Office DBP, mmHg	79.1 ± 10.5	79.9 ± 10.1	78.3 ± 10.9
Serum potassium, mEq/L	4.2 ± 0.4 *n *= 278	4.3 ± 0.4 *n *= 145	4.2 ± 0.4 *n *= 133
<4.5	209 (74.9)	105 (72.4)	104 (77.6)
≥4.5	69 (24.7)	40 (27.6)	29 (21.6)
eGFR_creat_, mL/min/1.73 m^2^	61.7 ± 18.2 *n *= 278	65.1 ± 19.8 *n *= 145	58.0 ± 15.4 *n *= 133
30 to <60	168 (60.2)	79 (54.5)	89 (66.4)
≥60	110 (39.4)	66 (45.5)	44 (32.8)
UACR, mg/gCr	144.1 ± 413.4 *n *= 278	174.1 ± 542.3 *n *= 145	111.3 ± 189.1 *n *= 133
<30	153 (54.8)	79 (54.5)	74 (55.2)
≥30	125 (44.8)	66 (45.5)	59 (44.0)
NT-proBNP, pg/mL	102.9 ± 156.8 *n *= 267	87.4 ± 117.1 *n *= 140	119.9 ± 190.4 *n *= 127
<125	215 (77.1)	115 (79.3)	100 (74.6)
≥125	52 (18.6)	25 (17.2)	27 (20.1)
Plasma aldosterone concentration, pg/mL	57.3 ± 38.0 *n *= 258	51.4 ± 40.0 *n *= 138	64.1 ± 34.4 *n *= 120
<120	246 (88.2)	131 (90.3)	115 (85.8)
≥120	12 (4.3)	7 (4.8)	5 (3.7)
Plasma renin activity, ng/mL/h	4.3 ± 9.6 *n *= 263	5.1 ± 11.7 *n *= 139	3.5 ± 6.3 *n *= 124
<1.0	82 (29.4)	42 (29.0)	40 (29.9)
≥1.0	181 (64.9)	97 (66.9)	84 (62.7)

Data are *n* (%) or mean ± standard deviation

*BMI* body mass index, *CCB* calcium channel blocker, *DBP* diastolic blood pressure, *DPP-4* dipeptidyl peptidase-4, *eGFR*_creat_ estimated glomerular filtration rate (creatinine-based), *GLP1* glucagon-like peptide-1, *NT-proBNP* N-terminal prohormone of brain natriuretic peptide, *RAS* renin–angiotensin system, *SBP* systolic blood pressure, *SGLT2i* sodium–glucose cotransporter-2 inhibitor, *UACR* urine albumin-to-creatinine ratio

### Antihypertensive effects

**Fig. 1:**
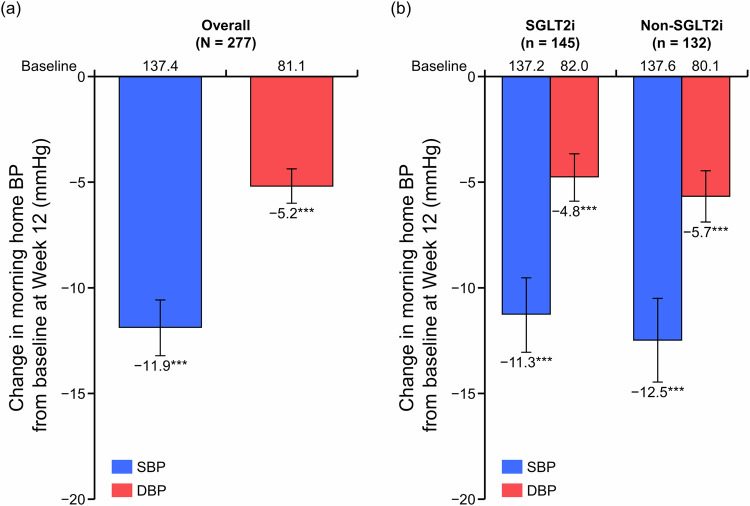
Changes in morning home BP in the overall population (**a**) and in the SGLT2i and non-SGLT2i subgroups (**b**) (full analysis set). Mean; error bars denote 95% confidence interval. ***P < 0.001 vs baseline. *BP* blood pressure, *DBP* diastolic BP, *SBP* systolic BP, *SGLT2i* sodium–glucose cotransporter-2 inhibitor

The changes in morning home BP, bedtime home BP, and office BP from baseline to Week 12 in the overall population and in the SGLT2i subgroups are shown in Fig. 1, Supplementary Fig. 1, and Supplementary Table 3. A significant change in morning home SBP/DBP from baseline to Week 12 was shown in the overall population (mean change: −11.9/−5.2 mmHg, both *P* < 0.001; Fig. 1a). This reduction in BP was consistent in both SGLT2i and non-SGLT2i subgroups (mean change: −11.3/−4.8 and −12.5/−5.7 mmHg, respectively, all *P* < 0.001; Fig. 1b). Similar findings were observed in bedtime home and office SBP/DBP (Supplementary Fig. 1a–d). The results in the FAS were consistent with those in the PPS (Supplementary Table 4).

**Fig. 2:**
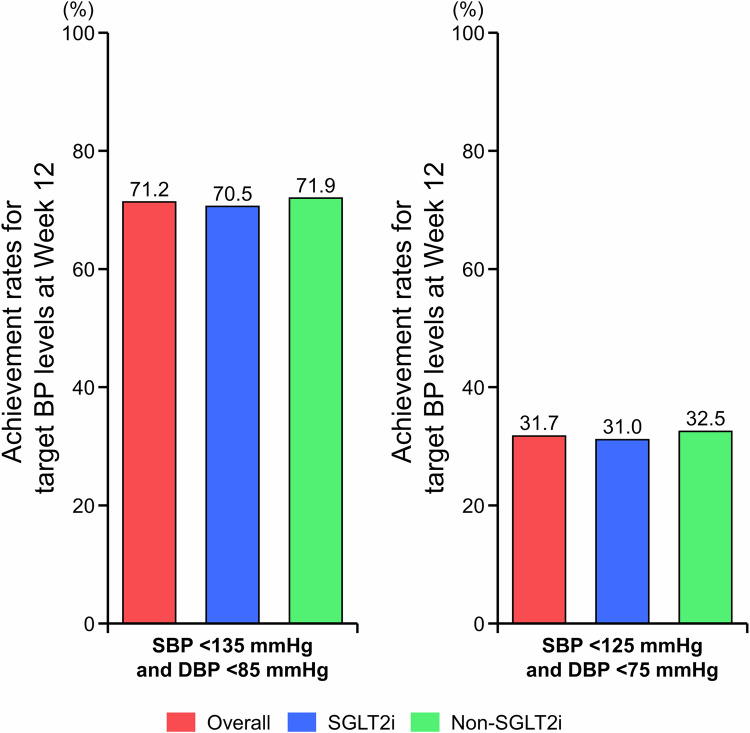
Achievement rates (morning home BP) for target BP levels (SBP/DBP < 135/85 mmHg and SBP/DBP < 125/75 mmHg) at Week 12 in the overall population and in the SGLT2i and non-SGLT2i subgroups (full analysis set). *BP* blood pressure, *DBP* diastolic BP, *SBP* systolic BP, *SGLT2i* sodium–glucose cotransporter-2 inhibitor

Achievement rates for target BP levels at Week 12 in the FAS are shown in Fig. 2, Supplementary Fig. 2, and Supplementary Table 5. The proportions of patients who achieved target home SBP/DBP < 135/85 mmHg, SBP < 135, and DBP < 85 mmHg in the overall population were 71.2%, 79.8%, and 81.5%, respectively. Similar achievement rates for target home SBP/DBP < 135/85 mmHg, target home SBP < 135, and target home DBP < 85 mmHg were observed irrespective of status of SGLT2i use: SGLT2i subgroup, 70.5%, 79.1%, and 80.6%; and non-SGLT2i subgroup, 71.9%, 80.7%, and 82.5%, respectively. Similar results were observed in the PPS (Supplementary Table 6).

### Effects on UACR and NT-proBNP

**Fig. 3:**
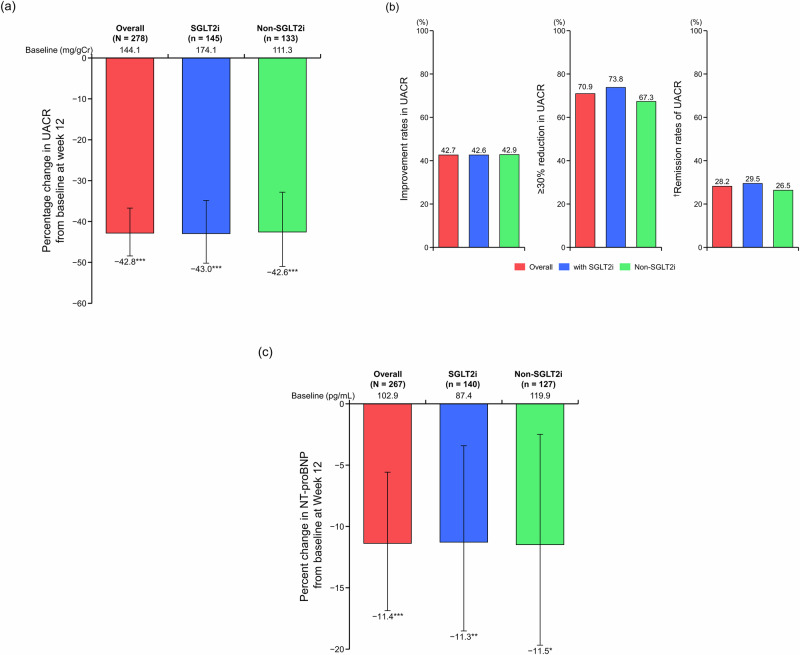
Percentage change in UACR from baseline to Week 12 (**a**), improvement rates of UACR (**b**), and percentage change in NT-proBNP from baseline to Week 12 (**c**) (full analysis set). Mean; error bars denote 95% confidence interval. For panel a, data include only patients with UACR A2 or A3 at baseline. ^†^Transition to UACR < 30 mg/gCr (UACR A1) from UACR 30– < 300 mg/gCr (UACR A2) or UACR ≥ 300 mg/gCr (UACR A3), along with a ≥30% reduction in UACR from baseline, *P < 0.05 vs baseline. **P < 0.01 vs baseline. ***P < 0.001 vs baseline. *NT-proBNP* N-terminal pro-B-type natriuretic peptide, *SGLT2i* sodium–glucose cotransporter-2 inhibitor, *UACR* urine albumin-to-creatinine ratio

The UACR significantly improved from baseline to Week 12 in the overall population and the SGLT2i subgroups (percentage change in geometric mean from baseline: −42.8% in the overall population and −43.0% and −42.6% in the SGLT2i and non-SGLT2i subgroups, respectively, all *P* < 0.001; Fig. 3a and Supplementary Table 7. The improvement rates of UACR, proportion of patients with ≥30% reduction in UACR, and UACR remission rates are shown in Fig. 3b and Supplementary Table 8. Among patients in the overall population, the improvement rate of UACR was 42.7%, 70.9% of patients had a ≥30% reduction in UACR, and 28.2% of patients achieved UACR remission in the A2 + A3 subcohort. Improvement rates of UACR, the proportion of patients with ≥30% reduction in UACR, and UACR remission rates were similar between the SGLT2i and non-SGLT2i subgroups: the respective improvement rates of UACR were 42.6% and 42.9%; 73.8% and 67.3% achieved a ≥30% reduction in UACR; and 29.5% and 26.5% achieved UACR remission in the A2 + A3 subcohort. Similar results were observed in the PPS (Supplementary Table 9). NT-proBNP significantly decreased from baseline to Week 12 (Fig. 3c and Supplementary Table 7).

### Safety

In the overall population, the incidence of TEAEs was 36.0%; serious TEAEs, 1.8%; and ADRs, 11.0%. There were no serious ADRs (Table 2). These results were similar between SGLT2i subgroups. The most frequent ADRs in the overall population were hyperkalemia (2.5%) and increased blood potassium (2.1%).

**Table 2:** Safety data (safety analysis set)

	Overall *N *= 283	SGLT2i *n *= 148	Non-SGLT2i *n *= 135
Any TEAE	102 (36.0)	52 (35.1)	50 (37.0)
Serious TEAEs	5 (1.8)	2 (1.4)	3 (2.2)
Cellulitis	1 (0.4)	1 (0.7)	0 (0.0)
Large intestine polyp	1 (0.4)	1 (0.7)	0 (0.0)
Diabetes mellitus	1 (0.4)	0 (0.0)	1 (0.7)
Atrial fibrillation	1 (0.4)	0 (0.0)	1 (0.7)
Upper limb fracture	1 (0.4)	0 (0.0)	1 (0.7)
Glaucoma surgery	1 (0.4)	0 (0.0)	1 (0.7)
Cataract operation	1 (0.4)	0 (0.0)	1 (0.7)
Any ADR	31 (11.0)	15 (10.1)	16 (11.9)
Hyperkalemia	7 (2.5)	3 (2.0)	4 (3.0)
Blood potassium increased	6 (2.1)	1 (0.7)	5 (3.7)
Dizziness	5 (1.8)	4 (2.7)	1 (0.7)
Dizziness postural	2 (0.7)	2 (1.4)	0 (0.0)
Headache	1 (0.4)	1 (0.7)	0 (0.0)
Dermal cyst	1 (0.4)	0 (0.0)	1 (0.7)
Rash	1 (0.4)	0 (0.0)	1 (0.7)
Blood pressure decreased	1 (0.4)	0 (0.0)	1 (0.7)
Glomerular filtration rate decreased	1 (0.4)	1 (0.7)	0 (0.0)
Hepatic enzyme increased	1 (0.4)	1 (0.7)	0 (0.0)
Salivary gland neoplasm	1 (0.4)	0 (0.0)	1 (0.7)
Eyelid edema	1 (0.4)	0 (0.0)	1 (0.7)
Tinnitus	1 (0.4)	0 (0.0)	1 (0.7)
Palpitations	1 (0.4)	0 (0.0)	1 (0.7)
Liver disorder	1 (0.4)	1 (0.7)	0 (0.0)
Feeling abnormal	1 (0.4)	1 (0.7)	0 (0.0)
Serious ADRs	0 (0.0)	0 (0.0)	0 (0.0)

Data are *n* (%)

*ADR* adverse drug reaction, *SGLT2i* sodium–glucose cotransporter-2 inhibitor, *TEAE* treatment-emergent adverse event

**Fig. 4:**
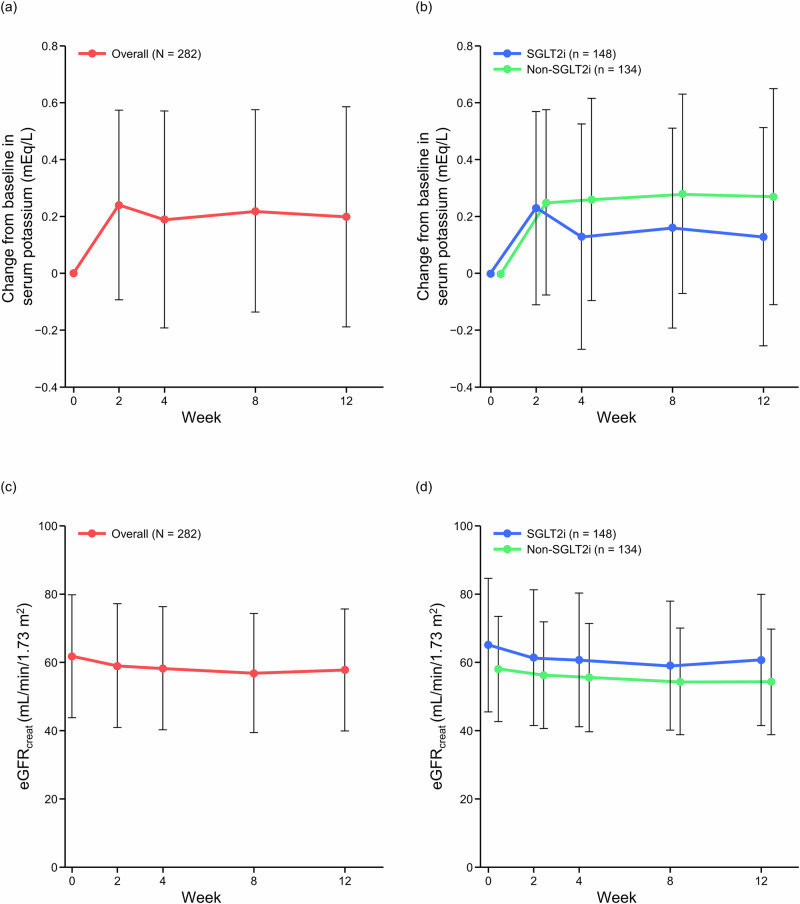
Change from baseline in serum potassium (**a**, **b**), and change in eGFR_creat_ (**c**, **d**) in the overall population and in the SGLT2i subgroups (safety analysis set). Mean; error bars denote 95% confidence interval. N indicates number of patients at baseline. *eGFR*_*creat*_ estimated glomerular filtration rate (creatinine-based), *SGLT2i* sodium–glucose cotransporter-2 inhibitor

Serum potassium levels increased after starting esaxerenone treatment up to Week 2, regardless of whether SGLT2is were used (Fig. 4a, b, Supplementary Fig. 3a, b, and Supplementary Table 10). Thereafter, serum potassium levels decreased up to Week 4 in the SGLT2i subgroup and remained stable up to Week 12. In the non-SGLT2i subgroup, serum potassium levels remained stable from Week 2 up to Week 12. The incidence of serum potassium ≥5.5 mEq/L was 3.5% (10/283 patients) in the overall population. The incidence of serum potassium ≥5.5 mEq/L was numerically lower in the SGLT2i subgroup than the non-SGLT2i subgroup (2.0% [3/148] vs 5.2% [7/135]); however, no statistical tests were performed (Supplementary Table 11).

In the overall population, eGFR_creat_ decreased after starting esaxerenone treatment up to Week 2, and thereafter remained stable up to Week 12 (Fig. 4c). Similar tendencies were observed in both SGLT2i subgroups, but baseline eGFR_creat_ was lower in the non-SGLT2i subgroup (Fig. 4d).

## Discussion

This pooled subanalysis aimed to evaluate the efficacy, organ-protective effects, and safety of esaxerenone in hypertensive patients with T2DM, with and without concomitant SGLT2i therapy in a clinical setting. The major findings of this study were that esaxerenone had a significant BP-lowering effect, improved albuminuria, and reduced NT-proBNP levels, regardless of SGLT2i use, indicating its renoprotective and cardioprotective effects as well as anti-hypertensive effects. The safety profile of esaxerenone was consistent, regardless of SGLT2i use, and concomitant use of an SGLT2i may help further enhance its safety profile regarding the mitigation of hyperkalemia risk in hypertensive patients with T2DM.

Of note, the consistent beneficial effects observed with esaxerenone across both subgroups in our study might suggest that the use of MRBs from an earlier stage in the clinical course could provide additional advantages, although further confirmatory data are needed. Moreover, in patients whose BP remains uncontrolled despite the addition of RAS inhibitor, CCB, and SGLT2i, enhanced volume management through MR blockade may help achieve more robust BP lowering. Conversely, in patients who are not receiving an SGLT2i, a relatively small degree of MR blockade may more readily reduce fluid volume and thus facilitate BP control, potentially reflecting heightened sensitivity to MR blockade in this population. SGLT2is exert their effects via the enhancement of glucose excretion through the urine while inducing natriuresis (sodium loss) and diuresis (fluid loss) [39]. In contrast, the mechanism underlying the antihypertensive effects of esaxerenone is the inhibition of sodium retention and consequent fluid overload, both of which are vital contributors to its antihypertensive effects. Therefore, the two drug classes exert their effects simultaneously without antagonizing each other, as they work via distinct biochemical pathways.

### Antihypertensive effects

Consistent with previous studies [26, 37], esaxerenone showed significant antihypertensive effects regardless of SGLT2i use. These findings align with previous analyses involving esaxerenone (the combined J308/J309 study analysis [37], EAGLE-DH [26], EARLY-NH [23], ESES-LVH [24], ENaK [25], and EXCITE-HT [40]) and reinforce that neither T2DM nor SGLT2i use adversely affect the BP-lowering efficacy of esaxerenone [38]. Notably, the antihypertensive effect of esaxerenone in patients with T2DM demonstrated in this study was numerically higher than that of finerenone in the FIDELIO-DKD study in patients with CKD and diabetes [16, 18]. However, direct comparisons are difficult and should be interpreted with caution because of the different types of SGLT2i used, disease characteristics of the included patients, baseline BP values and kidney function, and basal antihypertensive use. A key finding of the recently published CONFIDENCE trial, which evaluated combination therapy with the SGLT2i empagliflozin plus finerenone in patients with CKD and T2DM, was that the combination of both drugs significantly reduced UACR compared with treatment with either alone [41]. This supports the theory that targeting multiple pathophysiologic pathways simultaneously may have an additive effect in the management of kidney complications associated with diabetes. Similarly, the EXCITE-HT study [40] showed that initiating esaxerenone in patients already taking SGLT2i and basal antihypertensive agents has the potential to provide additional antihypertensive and UACR improvement, without a significant increase in adverse effects. These findings point to a paradigm shift in hypertension management strategies for patients with T2DM, suggesting that early intervention with combination therapy will become increasingly important to maximize the protection of kidney and cardiovascular function.

Notably, in the present study, the five studies on which it was based targeted patients with insufficient BP control despite the use of RAS inhibitors, ARBs, or CCBs, indicating that esaxerenone provides favorable antihypertensive effects regardless of SGLT2i use in such patients.

### UACR

Approximately 80% of patients received RAS inhibitors alone or combined with a CCB for at least 1 month before starting esaxerenone therapy, likely placing them at steady-state responses for UACR improvement from these agents. Therefore, the UACR-lowering effect observed in this analysis can be attributed primarily to esaxerenone. This finding is consistent with the J308/J309 combined analysis [37] and the EAGLE-DH study [26]. The observed renoprotective effect, reflected by the reduction in albuminuria and improvement in UACR classification, is clinically meaningful. Furthermore, these effects align with data from finerenone studies showing UACR reductions and improved kidney and cardiovascular outcomes [16, 18, 42]. Given this, esaxerenone, a selective MRB that works by inhibiting the effects of aldosterone, may potentially mitigate aldosterone-induced kidney damage, although further long-term study is required to verify the effects of esaxerenone on kidney and cardiovascular outcomes.

### Serum potassium

Serum potassium levels increased up to Week 2 after starting esaxerenone treatment but stabilized thereafter through Week 12 in both subgroups. The incidence of serum potassium ≥5.5 mEq/L was numerically lower among patients receiving concomitant SGLT2i therapy, mirroring previous reports indicating that SGLT2is can lessen the risk of hyperkalemia associated with MRBs [34–36]. SGLT2is may provide potential protection against hyperkalemia through a multifaceted mechanism of the promotion of natriuresis, regulation of potassium processing in the renal tubules (inhibiting sodium reabsorption), and by reducing RAS activity. This mechanism may allow for the concomitant use of esaxerenone, which may improve patient safety and therapeutic outcomes. Notably, in the present study, the total number of patients with serum potassium levels ≥5.5 mEq/L was low compared with previous clinical trials of esaxerenone (7.9% in a previous pooled analysis [43]). This may be partly because in the five studies included in this pooled analysis, esaxerenone dosing was adjusted at the treating physician’s discretion according to the patient’s condition (antihypertensive effect and serum potassium level) and the package insert [44]. The package insert for esaxerenone specifies that patients with diabetes and moderate kidney dysfunction (eGFR 30–60 mL/min/1.73 m^2^ and albuminuria) should receive half-doses of esaxerenone. That is, it is possible that dose reductions for high-risk patients and thorough monitoring in actual clinical conditions contributed to the lower incidence of hyperkalemia observed in this study compared with clinical trials [21]. Furthermore, although eGFR_creat_ declined during the first 2 weeks of esaxerenone treatment and then stabilized, baseline eGFR_creat_ was lower in the non-SGLT2i subgroup. Overall, esaxerenone provided sustained 24-h BP control and improvements in albuminuria without raising safety concerns when dosed and monitored according to the package insert.

### Clinical implications

Importantly, finerenone is not indicated for its antihypertensive effect (i.e. the treatment of hypertension) but is indicated for the treatment of CKD complicated by T2DM. In contrast, esaxerenone is indicated for the treatment of hypertension. Therefore, when selecting MRBs to treat hypertension, it is recommended that esaxerenone is administered instead of finerenone. The draft JSH 2025 guidelines [45] recommend MRBs as second-line therapy and permit the administration of esaxerenone as an additional antihypertensive agent when there is an inadequate antihypertensive effect with ARB or CCB monotherapy. The 2024 Japanese Clinical Practice Guidelines for the diagnosis and treatment of CKD [46] recommend SGLT2is as first-line agents for CKD complicated by T2DM, and RAS-based inhibitors for hypertension with proteinuria. Additionally, nonsteroidal MRBs are recommended for persistent albuminuria. Finerenone is recommended if serum potassium levels are normal and only the improvement of albuminuria is needed, while esaxerenone is recommended if both improvement of albuminuria and hypertension are needed.

### Limitations

The limitations are generally similar to those of the primary analysis [38]. First, esaxerenone was used in combination with other antihypertensive agents (primarily a second or third agent), meaning its efficacy as monotherapy was not assessed. However, previous studies demonstrate that esaxerenone monotherapy exerts notable BP-lowering effects [47]. Second, the antihypertensive and UACR-improving effects of esaxerenone reported here are for the first 12 weeks after administration, and the effects of esaxerenone on long-term outcomes such as cardiovascular and kidney events were not confirmed in this study. Third, antihypertensive effects of esaxerenone in relation to specific CKD stage (kidney function) were not assessed, leaving it unclear whether the improvement in UACR was entirely BP-dependent or partially independent of BP control. Fourth, the study population was limited to Japanese patients, and the results may not be generalizable to other populations. Fifth, there was heterogeneity among the five clinical studies on which the present study was based in terms of research objectives, BP targets, and concomitant medication parameters. Nevertheless, in our study, data were pooled using the following common criteria: (1) patients with defined hypertension plus T2DM; (2) available data for indicators including BP, UACR, and NT-proBNP; and (3), available results at the 12-week time point. Finally, patient backgrounds between those prescribed and not prescribed SGLT2is may vary, and the proportion of patients with T2DM in the EARLY-NH, ESES-LVH, and ENaK studies was low [23–25].

## Conclusion

This pooled subanalysis indicated that esaxerenone significantly lowered morning home, bedtime home, and office BP in hypertensive patients with T2DM, regardless of SGLT2i use. Esaxerenone also reduced UACR and improved NT-proBNP levels in both subgroups, underscoring its kidney and cardioprotective effects. The overall safety was comparable between subgroups, and the incidence of serum potassium ≥5.5 mEq/L was numerically lower among those receiving SGLT2i. These findings suggest that concomitant SGLT2i therapy may help mitigate the risk of hyperkalemia in hypertensive patients with T2DM who are treated with esaxerenone.

Supplementary information is available at *Hypertension Research’s* website.

## Supplementary information

Supplementary materials

## Data Availability

The anonymized data underlying the results presented in this manuscript may be made available to researchers upon submission of a reasonable request to the corresponding author. The decision to disclose the data will be made by the corresponding author and the funder, Daiichi Sankyo Co., Ltd. Data disclosure can be requested for 36 months from article publication.
