# Supplementary material for: Efficacy and safety of esaxerenone with and without sodium–glucose cotransporter-2 inhibitor use in hypertensive patients with type 2 diabetes mellitus: a pooled analysis of five clinical studies
Source: Hypertens Res. 2025 Sep 1;48(11):2924–37. doi: 10.1038/s41440-025-02347-0 (PMC12586164; doi:10.1038/s41440-025-02347-0)
Supplement: Supplementary file 1 — Supplementary materials [file 41440_2025_2347_MOESM1_ESM.docx]

**Supplementary Materials**

**Supplemental Figures**

**
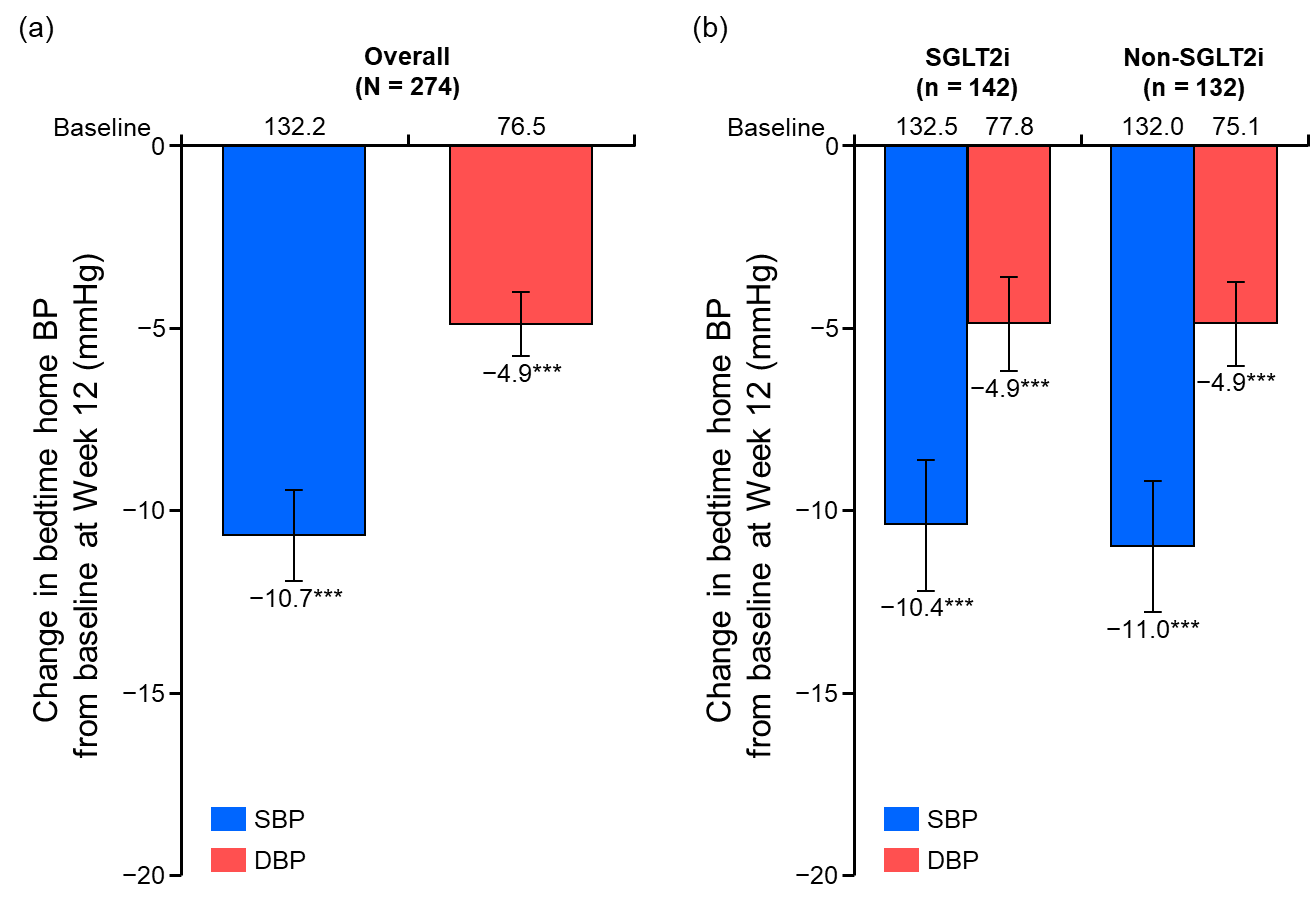
**

**
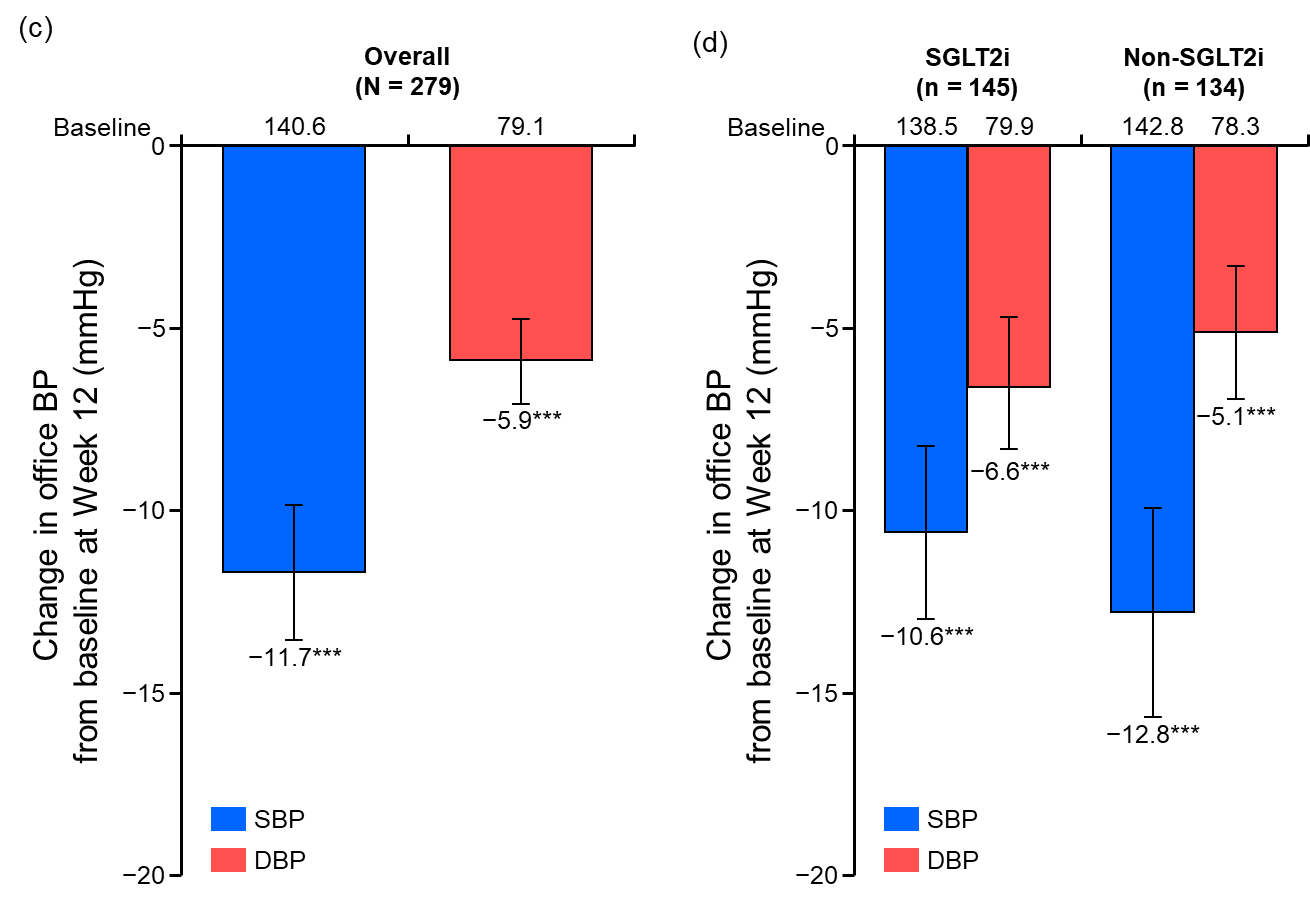
**

**Supplementary Figure 1.** Changes in bedtime home BP (**a**, **b**), and office BP (**c**, **d**) in the overall population and in the SGLT2i and non-SGLT2i subgroups (full analysis set)

Mean; error bars denote 95% confidence interval

****P* <0.001 vs baseline

Abbreviations: BP, blood pressure; DBP, diastolic BP; SBP, systolic BP; SGLT2i, sodium–glucose cotransporter-2 inhibitor

**
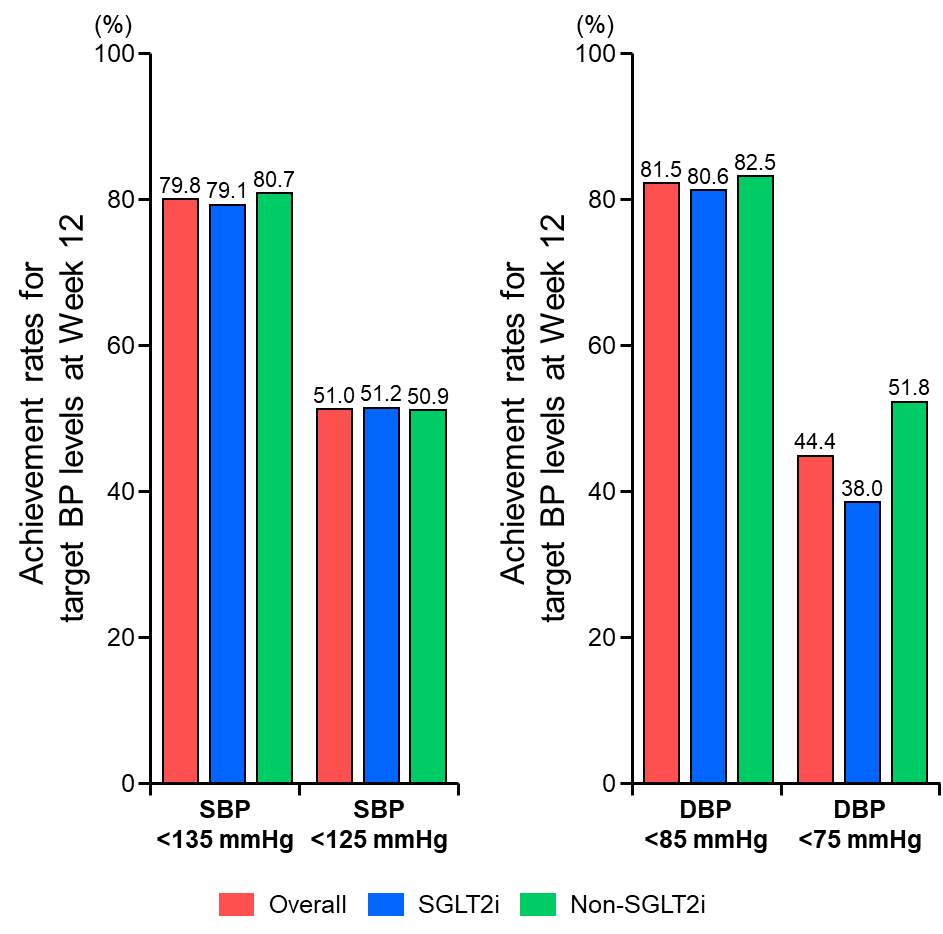
**

**Supplementary Figure 2.** Achievement rates (morning home BP) for target BP levels (SBP <135 mmHg, SBP <125 mmHg, DBP <85 mmHg, and DBP <75 mmHg) at Week 12 in the overall population and in the SGLT2i and non-SGLT2i subgroups (full analysis set)

Abbreviations: BP, blood pressure; DBP, diastolic BP; SBP, systolic BP; SGLT2i, sodium–glucose cotransporter-2 inhibitor

**
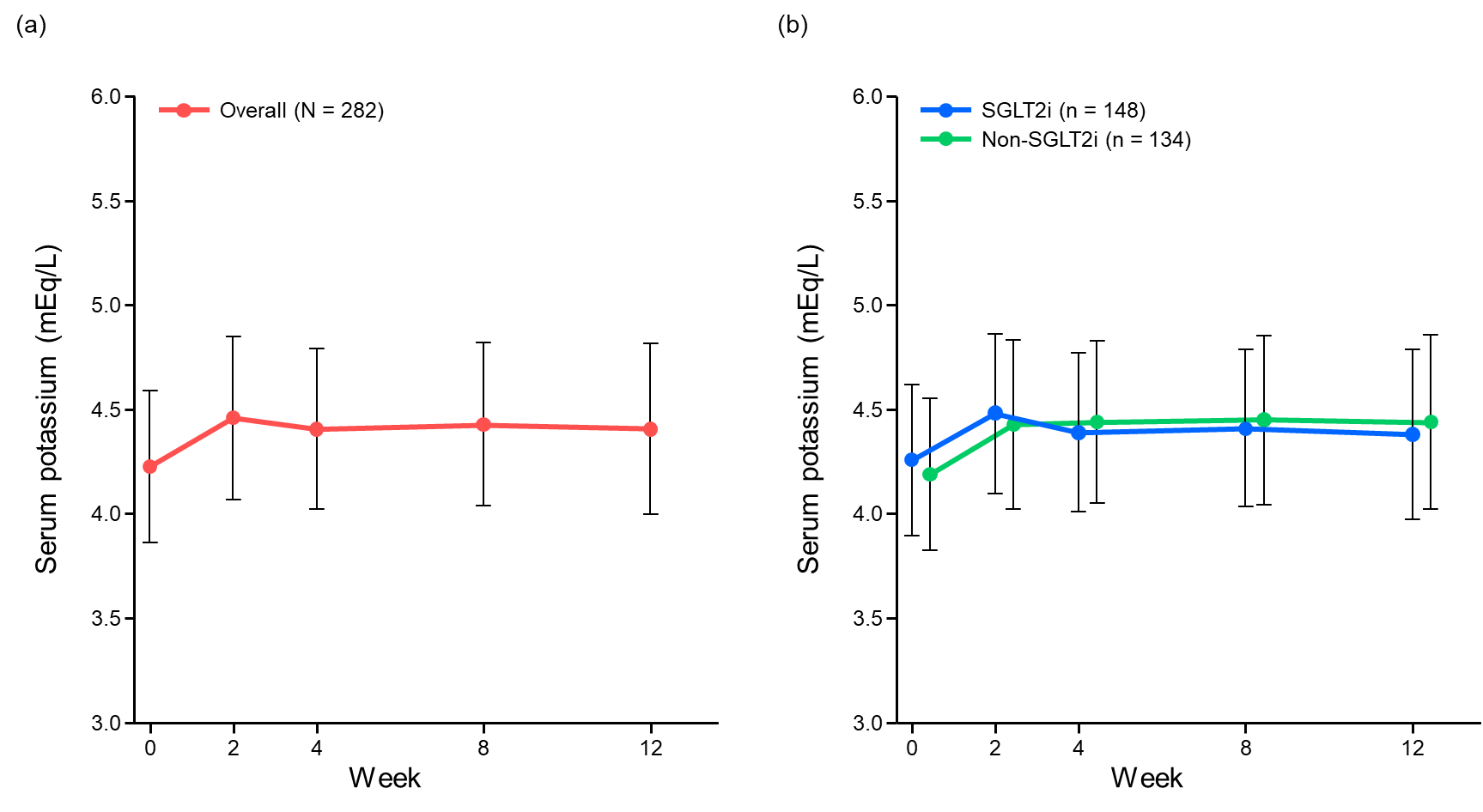
**

**Supplementary Figure 3.** Change in serum potassium in the overall population (**a**) and in the SGLT2i subgroups (**b**) (safety analysis set)

Mean; error bars denote 95% confidence interval

N indicates number of patients at baseline.

SGLT2i, sodium–glucose cotransporter-2 inhibitor

**Supplementary Table 1.** Target population of each of the studies included in this pooled subanalysis [38]

| **Study** | **Target population** | **FAS, N** | **Mean morning home SBP/DBP, mmHg** | | | **UACR** | | **T2DM,**  **n (%)** | **Incidence of serum potassium**  **≥5.5 mEq/L** |
| --- | --- | --- | --- | --- | --- | --- | --- | --- | --- |
|  |  |  | **Baseline** | **Week 12** | **EOT** | **Mean at baseline** | **<30 / 30 to <300**  **/ ≥300 mg/gCr,**  **n (%)** |  |  |
| EX-DKD [22] | Hypertensive patients with DKD | 109 | 135.6/75.9 | −12.0/−5.2 | −11.6/−5.2 | 184.0 | 45 (41.3)/ 41 (37.6)/ 23 (21.1) | 109 (100.0) | 3 (2.7) |
| EARLY-NH [23] | Patients with nocturnal hypertension | 93 | 143.8/86.7 | −11.9/−4.2 | −12.2/−4.9 | 110.4 | 65 (69.9)/ 19 (20.4)/ 9 (9.7) | 33 (35.5) | 11 (10.9) |
| ESES-LVH^a^ [24] | Hypertensive patients with LVH | 58 | 142.8/85.0 | −10.5/−4.8 | −11.9/−5.0 | NC | NC | 11 (19.0) | 3 (5.0) |
| ENaK [25] | Patients with essential hypertension | 126 | 136.7/88.0 | −12.3/−6.7 | −11.9/−6.4 | 34.6 | 101 (81.5)/ 20 (16.1)/ 3 (2.4) | 33 (26.2) | 9 (7.1) |
| EAGLE-DH^a^ [26] | Hypertensive patients with diabetes taking SGLT2i | 93 | 136.4/82.3 | −11.8/−5.1 | −12.9/−5.7 | 145.3 | 58 (62.4)/ 28 (30.1)/ 7 (7.5) | 93 (100.0) | 1 (1.1) |

^a^The dosing period for ESES-LVH and EAGLE-DH was 24 weeks

Abbreviations: DBP, diastolic blood pressure; DKD, diabetic kidney disease; EOT, end of treatment; FAS, full analysis set; LVH, left ventricular hypertrophy; NC, not calculated; SBP, systolic blood pressure; SGLT2i, sodium–glucose cotransporter-2 inhibitor; T2DM, type 2 diabetes mellitus; UACR, urine albumin-to-creatinine ratio

**Supplementary Table 2.** Patient characteristics (per-protocol set)

| **Characteristic** | **Overall**  **N = 253** | **SGLT2i**  **n = 132** | **Non-SGLT2i**  **n = 121** |
| --- | --- | --- | --- |
| Sex, male | 148 (58.5) | 89 (67.4) | 59 (48.8) |
| Age, years | 68.5 ± 9.8 | 67.1 ± 9.9 | 70.0 ± 9.6 |
| ≥65 | 182 (71.9) | 87 (65.9) | 95 (78.5) |
| BMI, kg/m^2^ | 26.2 ± 4.1  n = 252 | 27.3 ± 4.1  n = 131 | 25.1 ± 3.8  n = 121 |
| ≥25 | 150 (59.3) | 90 (68.2) | 60 (49.6) |
| Initial esaxerenone dose, mg/day | 1.6 ± 0.6 | 1.7 ± 0.6 | 1.5 ± 0.5 |
| 1.25 | 180 (71.1) | 86 (65.2) | 94 (77.7) |
| 2.5 | 73 (28.9) | 46 (34.8) | 27 (22.3) |
| Final esaxerenone dose, mg/day | 2.5 ± 1.4 | 2.7 ± 1.4 | 2.3 ± 1.3 |
| 1.25 | 99 (39.1) | 41 (31.1) | 58 (47.9) |
| 2.5 | 103 (40.7) | 59 (44.7) | 44 (36.4) |
| 5 | 51 (20.2) | 32 (24.2) | 19 (15.7) |
| Dose escalation from the first dose of esaxerenone to 12 weeks | 109 (43.1) | 61 (46.2) | 48 (39.7) |
| Current smoker | 37 (14.6) | 23 (17.4) | 14 (11.6) |
| Alcohol use | 94 (37.2) | 57 (43.2) | 37 (30.6) |
| Complications | 251 (99.2) | 131 (99.2) | 120 (99.2) |
| Dyslipidemia | 203 (80.2) | 107 (81.1) | 96 (79.3) |
| Hyperuricemia | 66 (26.1) | 34 (25.8) | 32 (26.4) |
| Heart failure | 21 (8.3) | 15 (11.4) | 6 (5.0) |
| Disease duration of hypertension, years | 10.9 ± 8.4  n = 176 | 10.9 ± 8.3  n = 95 | 10.8 ± 8.5  n = 81 |
| Basal antihypertensive agents |  |  |  |
| RAS inhibitor | 102 (40.3) | 46 (34.8) | 56 (46.3) |
| CCB | 40 (15.8) | 24 (18.2) | 16 (13.2) |
| Both RAS inhibitor/CCB | 111 (43.9) | 62 (47.0) | 49 (40.5) |
| Diabetes drug class |  |  |  |
| SGLT2i | 132 (52.2) | 132 (100.0) | 0 (0.0) |
| Biguanide | 106 (41.9) | 49 (37.1) | 57 (47.1) |
| Thiazolidinedione | 12 (4.7) | 5 (3.8) | 7 (5.8) |
| Sulfonylurea | 39 (15.4) | 18 (13.6) | 21 (17.4) |
| Glinide | 22 (8.7) | 9 (6.8) | 13 (10.7) |
| DPP-4 inhibitor | 147 (58.1) | 76 (57.6) | 71 (58.7) |
| Alpha-glucosidase inhibitor | 26 (10.3) | 6 (4.5) | 20 (16.5) |
| Insulin | 22 (8.7) | 8 (6.1) | 14 (11.6) |
| GLP1 agonist | 13 (5.1) | 6 (4.5) | 7 (5.8) |
| Number of oral diabetes medications |  |  |  |
| 1 | 65 (25.7) | 31 (23.5) | 34 (28.1) |
| 2 | 79 (31.2) | 39 (29.5) | 40 (33.1) |
| ≥3 | 90 (35.6) | 62 (47.0) | 28 (23.1) |
| None | 19 (17.5) | 0 (0.0) | 19 (15.7) |
| Morning home SBP, mmHg | 137.2 ± 11.7  n = 251 | 137.4 ± 11.3  n = 132 | 137.0 ± 12.2  n = 119 |
| Morning home DBP, mmHg | 80.8 ± 10.6  n = 251 | 81.8 ± 9.7  n = 132 | 79.7 ± 11.5  n = 119 |
| Bedtime home SBP, mmHg | 132.1 ± 13.0  n = 248 | 133.0 ± 11.9  n = 129 | 131.2 ± 14.1  n = 119 |
| Bedtime home DBP, mmHg | 76.2 ± 11.2  n = 248 | 77.8 ± 10.6  n = 129 | 74.4 ± 11.6  n = 119 |
| Office SBP, mmHg | 140.1 ± 13.9 | 137.6 ± 14.3 | 143.0 ± 12.9 |
| Office DBP, mmHg | 78.4 ± 10.3 | 79.0 ± 9.8 | 77.8 ± 10.9 |
| Serum potassium, mEq/L | 4.2 ± 0.3 | 4.3 ± 0.3 | 4.2 ± 0.3 |
| <4.5 | 188 (74.3) | 96 (72.7) | 92 (76.0) |
| ≥4.5 | 65 (25.7) | 36 (27.3) | 29 (24.0) |
| eGFR_creat_, mL/min/1.73 m^2^ | 60.4 ± 17.8 | 63.8 ± 19.7 | 56.8 ± 14.7 |
| <30 | 0 (0.0) | 0 (0.0) | 0 (0.0) |
| 30 to <60 | 163 (64.4) | 77 (58.3) | 86 (71.1) |
| ≥60 | 90 (35.6) | 55 (41.7) | 35 (28.9) |
| UACR, mg/gCr | 147.9 ± 432.0  n = 252 | 183.4 ± 567.6  n = 132 | 108.9 ± 189.8  n = 120 |
| <30 | 150 (59.3) | 78 (59.1) | 72 (59.5) |
| ≥30 | 102 (40.3) | 54 (40.9) | 48 (39.7) |
| NT-proBNP, pg/mL | 100.2 ± 152.0  n = 243 | 90.1 ± 121.5  n = 127 | 111.2 ± 179.5  n = 116 |
| <125 | 197 (77.9) | 103 (78.0) | 94 (77.7) |
| ≥125 | 46 (18.2) | 24 (18.2) | 22 (18.2) |
| Plasma aldosterone concentration, pg/mL | 58.6 ± 39.0  n = 235 | 52.6 ± 41.6  n = 126 | 65.6 ± 34.5  n = 109 |
| <120 | 222 (87.7) | 118 (89.4) | 104 (86.0) |
| ≥120 | 13 (5.1) | 8 (6.1) | 5 (4.1) |
| Plasma renin activity, ng/mL/h | 4.3 ± 9.7  n = 239 | 5.2 ± 12.0  n = 126 | 3.3 ± 6.1  n = 113 |
| <1.0 | 73 (28.9) | 36 (27.3) | 37 (30.6) |
| ≥1.0 | 166 (65.6) | 90 (68.2) | 76 (62.8) |

Data are n (%) unless otherwise indicated.

Abbreviations: BMI, body mass index; CCB, calcium channel blocker; DBP, diastolic blood pressure; DPP-4, dipeptidyl peptidase-4; eGFR_creat_, estimated glomerular filtration rate (creatinine-based); GLP1, glucagon-like peptide-1; NT-proBNP, N-terminal prohormone of brain natriuretic peptide; RAS, renin–angiotensin system; SBP, systolic blood pressure; SGLT2i, sodium–glucose cotransporter-2 inhibitor; UACR, urine albumin-to-creatinine ratio

**Supplementary Table 3.** Changes in morning home BP, bedtime home BP, and office BP (full analysis set)

|  | **Overall** | | | **SGLT2i** | | | **Non-SGLT2i** | | |
| --- | --- | --- | --- | --- | --- | --- | --- | --- | --- |
|  | **n** | **SBP, mmHg** | **DBP, mmHg** | **n** | **SBP, mmHg** | **DBP, mmHg** | **n** | **SBP, mmHg** | **DBP, mmHg** |
| Morning home BP, mmHg |  |  |  |  |  |  |  |  |  |
| Baseline | 277 | 137.4 ± 11.7 | 81.1 ± 10.3 | 145 | 137.2 ± 11.2 | 82.0 ± 9.4 | 132 | 137.6 ± 12.2 | 80.1 ± 11.3 |
| Week 2 | 176 | 131.8 ± 12.8 | 79.0 ± 10.8 | 50 | 133.9 ± 12.6 | 80.8 ± 8.7 | 126 | 131.0 ± 12.8 | 78.3 ± 11.5 |
| Change from baseline  [95% CI] | 174 | −6.1 ± 8.9***  [−7.4, −4.7] | −1.8 ± 5.3***  [−2.6, −1.0] | 50 | −5.1 ± 6.6***  [−7.0, −3.2] | −1.2 ± 4.9  [−2.7, 0.2] | 124 | −6.5 ± 9.6***  [−8.2, −4.8] | −2.0 ± 5.4***  [−3.0, −1.1] |
| Week 4 | 263 | 129.4 ± 11.9 | 78.4 ± 10.0 | 140 | 129.8 ± 11.8 | 79.5 ± 9.2 | 123 | 129.0 ± 12.0 | 77.2 ± 10.8 |
| Change from baseline  [95% CI] | 262 | −8.1 ± 8.0***  [−9.0, −7.1] | −2.9 ± 4.8***  [−3.5, −2.3] | 140 | −7.7 ± 8.1***  [−9.0, −6.3] | −2.7 ± 5.0***  [−3.6, −1.9] | 122 | −8.5 ± 7.8***  [−9.9, −7.1] | −3.1 ± 4.6***  [−3.9, −2.3] |
| Week 8 | 237 | 127.2 ± 12.3 | 76.9 ± 10.0 | 122 | 128.6 ± 12.1 | 78.3 ± 9.4 | 115 | 125.7 ± 12.3 | 75.3 ± 10.4 |
| Change from baseline  [95% CI] | 236 | −10.4 ± 9.6***  [−11.7, −9.2] | −4.2 ± 5.6*** [−4.9, −3.5] | 122 | −9.1 ± 8.9***  [−10.6, −7.5] | −3.5 ± 5.2***  [−4.4, −2.5] | 114 | −11.9 ± 10.1***  [−13.8, −10.0] | −5.0 ± 5.8***  [−6.1, −3.9] |
| Week 12 | 243 | 125.8 ±12.2 | 76.1 ± 9.8 | 129 | 126.6 ± 11.9 | 77.5 ± 8.9 | 114 | 124.9 ± 12.5 | 74.6 ± 10.5 |
| Change from baseline  [95% CI] | 242 | −11.9 ± 10.4***  [−13.2, −10.5] | −5.2 ± 6.5***  [−6.1, −4.4] | 129 | −11.3 ± 10.2***  [−13.0, −9.5] | −4.8 ± 6.4***  [−6.0, −3.7] | 113 | −12.5 ± 10.6***  [−14.5, −10.5] | −5.7 ± 6.5*** [−6.9, −4.5] |
| Bedtime home BP, mmHg |  |  |  |  |  |  |  |  |  |
| Baseline | 274 | 132.2 ± 13.3 | 76.5 ± 11.2 | 142 | 132.5 ± 12.6 | 77.8 ± 10.8 | 132 | 132.0 ± 14.1 | 75.1 ± 11.4 |
| Week 2 | 175 | 126.4 ± 13.2 | 74.2 ± 10.4 | 50 | 127.6 ± 13.1 | 75.4 ± 8.7 | 125 | 125.9 ± 13.2 | 73.7 ± 11.0 |
| Change from baseline  [95% CI] | 173 | −6.1 ± 8.0***  [−7.3, −4.9] | −1.8 ± 4.8***  [−2.5, −1.1] | 50 | −6.7 ± 8.3***  [−9.1, −4.4] | −2.8 ± 5.4***  [−4.3, −1.2] | 123 | −5.8 ± 7.8***  [−7.2, −4.4] | −1.4 ± 4.4***  [−2.2, −0.6] |
| Week 4 | 263 | 124.5 ± 13.3 | 73.8 ± 10.6 | 140 | 125.6 ± 12.4 | 75.1 ± 9.6 | 123 | 123.2 ± 14.2 | 72.3 ± 11.4 |
| Change from baseline  [95% CI] | 259 | −7.5 ± 8.4***  [−8.6, −6.5] | −2.8 ± 5.4***  [−3.4, −2.1] | 137 | −7.0 ± 8.8***  [−8.5, −5.5] | −2.8 ± 6.1***  [−3.9, −1.8] | 122 | −8.2 ± 8.0***  [−9.6, −6.7] | −2.7 ± 4.5***  [−3.5, −1.9] |
| Week 8 | 237 | 122.1 ± 13.4 | 71.9 ± 10.7 | 122 | 123.6 ± 12.7 | 73.5 ± 10.2 | 115 | 120.5 ± 13.9 | 70.2 ± 11.1 |
| Change from baseline  [95% CI] | 234 | −9.8 ± 9.9***  [−11.1, −8.5] | −4.2 ± 6.1***  [−5.0, −3.5] | 120 | −9.0 ± 10.7***  [−11.0, −7.1] | −3.8 ± 6.8***  [−5.0, −2.6] | 114 | −10.6 ± 8.9***  [−12.2, −8.9] | −4.7 ± 5.2***  [−5.7, −3.7] |
| Week 12 | 243 | 121.5 ± 12.7 | 71.8 ± 10.3 | 129 | 122.8 ± 11.6 | 73.4 ± 9.3 | 114 | 120.1 ± 13.8 | 70.1 ± 11.2 |
| Change from baseline  [95% CI] | 239 | −10.7 ± 9.9***  [−11.9, −9.4] | −4.9 ± 6.8***  [−5.8, −4.1] | 126 | −10.4 ± 10.1***  [−12.1, −8.6] | −4.9 ± 7.3***  [−6.2, −3.6] | 113 | −11.0 ± 9.6***  [−12.8, −9.2] | −4.9 ± 6.2***  [−6.1, −3.8] |
| Office BP, mmHg |  |  |  |  |  |  |  |  |  |
| Baseline | 279 | 140.6 ± 14.0 | 79.1 ± 10.5 | 145 | 138.5 ± 14.8 | 79.9 ± 10.1 | 134 | 142.8 ± 12.7 | 78.3 ± 10.9 |
| Week 2 | 108 | 135.1 ± 15.3 | 73.1 ± 9.6 | 28 | 133.6 ± 10.9 | 75.5 ± 8.5 | 80 | 135.7 ± 16.5 | 72.3 ± 9.9 |
| Change from baseline  [95% CI] | 108 | −9.5 ± 12.9***  [−11.9, −7.0] | −3.6 ± 7.9***  [−5.1, −2.1] | 28 | −9.9 ± 12.3***  [−14.7, −5.2] | −1.9 ± 6.2  [−4.3, 0.5] | 80 | −9.3 ± 13.1***  [−12.2, −6.4] | −4.2 ± 8.3***  [−6.0, −2.3] |
| Week 4 | 264 | 131.4 ± 14.9 | 75.2 ± 10.6 | 140 | 129.8 ± 14.2 | 76.1 ± 10.2 | 124 | 133.2 ± 15.6 | 74.3 ± 11.0 |
| Change from baseline  [95% CI] | 264 | −8.8 ± 14.1***  [−10.5, −7.1] | −3.8 ± 9.1***  [−4.9, −2.7] | 140 | −8.3 ± 14.2***  [−10.7, −5.9] | −3.8 ± 9.0***  [−5.3, −2.3] | 124 | −9.3 ± 14.0***  [−11.8, −6.9] | −3.8 ± 9.2*** [−5.5, −2.2] |
| Week 8 | 239 | 130.0 ± 14.4 | 73.9 ± 10.6 | 125 | 128.6 ± 13.7 | 74.5 ± 10.2 | 114 | 131.4 ± 15.1 | 73.2 ± 11.0 |
| Change from baseline  [95% CI] | 239 | −10.0 ± 13.7***  [−11.8, −8.3] | −5.0 ± 9.0***  [−6.1, −3.8] | 125 | −9.2 ± 12.4***  [−11.4, −7.0] | −5.0 ± 9.1***  [−6.6, −3.4] | 114 | −10.9 ± 15.0***  [−13.7, −8.1] | −4.9 ± 9.0***  [−6.6, −3.2] |
| Week 12 | 249 | 128.0 ± 14.3 | 73.2 ± 9.9 | 133 | 126.9 ± 13.3 | 73.3 ± 8.8 | 116 | 129.3 ± 15.3 | 73.1 ± 11.1 |
| Change from baseline  [95% CI] | 249 | −11.7 ± 14.8***  [−13.5, −9.8] | −5.9 ± 9.4*** [−7.0, −4.7] | 133 | −10.6 ± 13.9***  [−13.0, −8.3] | −6.6 ± 8.9***  [−8.1, −5.1] | 116 | −12.8 ± 15.7***  [−15.7, −10.0] | −5.1 ± 9.9***  [−6.9, −3.2] |

Data are mean ± standard deviation.

****P* <0.001 versus baseline, paired *t*-test.

Abbreviations: BP, blood pressure; CI, confidence interval; DBP, diastolic blood pressure; SBP, systolic blood pressure; SGLT2i, sodium–glucose cotransporter-2 inhibitor

**Supplementary Table 4.** Changes in morning home BP, bedtime home BP, and office BP (per protocol set)

|  | **Overall** | | | **SGLT2i** | | | **Non-SGLT2i** | | |
| --- | --- | --- | --- | --- | --- | --- | --- | --- | --- |
|  | **n** | **SBP, mmHg** | **DBP, mmHg** | **n** | **SBP, mmHg** | **DBP, mmHg** | **n** | **SBP, mmHg** | **DBP, mmHg** |
| Morning home BP, mmHg |  |  |  |  |  |  |  |  |  |
| Baseline | 251 | 137.2 ± 11.7 | 80.8 ± 10.6 | 132 | 137.4 ± 11.3 | 81.8 ± 9.7 | 119 | 137.0 ± 12.2 | 79.7 ± 11.5 |
| Week 2 | 163 | 131.5 ± 12.9 | 78.7 ± 10.9 | 49 | 134.1 ± 12.7 | 80.9 ± 8.8 | 114 | 130.4 ± 12.8 | 77.7 ± 11.5 |
| Change from baseline  [95% CI] | 161 | −6.1 ± 8.7***  [−7.4, −4.7] | −1.8 ± 5.0***  [−2.6, −1.0] | 49 | −5.2 ± 6.7***  [−7.1, −3.2] | −1.1 ± 5.0  [−2.5, 0.3] | 112 | −6.5 ± 9.4***  [−8.2, −4.7] | −2.1 ± 5.1***  [−3.1, −1.2] |
| Week 4 | 238 | 129.4 ± 12.1 | 78.1 ± 10.2 | 126 | 130.0 ± 12.1 | 79.4 ± 9.4 | 112 | 128.6 ± 12.1 | 76.7 ± 10.9 |
| Change from baseline  [95% CI] | 237 | −7.8 ± 7.9***  [−8.8, −6.8] | −2.8 ± 4.8***  [−3.5, −2.2] | 126 | −7.5 ± 8.0***  [−8.9, −6.1] | −2.6 ± 5.1*** [−3.5, −1.7] | 111 | −8.2 ± 7.7***  [−9.7, −6.8] | −3.1 ± 4.4***  [−3.9, −2.3] |
| Week 8 | 214 | 127.1 ± 12.4 | 76.6 ± 10.3 | 110 | 128.7 ± 12.1 | 78.2 ± 9.6 | 104 | 125.3 ± 12.5 | 75.0 ± 10.7 |
| Change from baseline  [95% CI] | 213 | −10.2 ± 9.2***  [−11.5, −9.0] | −4.1 ± 5.4***  [−4.9, −3.4] | 110 | −9.0 ± 8.4***  [−10.6, −7.4] | −3.5 ± 5.1***  [−4.5, −2.5] | 103 | −11.6 ± 9.7***  [−13.5, −9.7] | −4.8 ± 5.6***  [−5.9, −3.7] |
| Week 12 | 219 | 125.6 ± 12.3 | 75.7 ± 9.9 | 116 | 126.8 ± 12.4 | 77.2 ± 9.2 | 103 | 124.3 ± 12.2 | 74.0 ± 10.3 |
| Change from baseline  [95% CI] | 218 | −11.7 ± 10.0***  [−13.1, −10.4] | −5.3 ± 6.1***  [−6.2, −4.5] | 116 | −11.1 ± 10.1***  [13.0, −9.3] | −4.9 ± 6.5***  [−6.1, −3.8] | 102 | −12.4 ± 9.9***  [−14.3, −10.5] | −5.8 ± 5.7***  [−6.9, −4.7] |
| Bedtime home BP, mmHg |  |  |  |  |  |  |  |  |  |
| Baseline | 248 | 132.1 ± 13.0 | 76.2 ± 11.2 | 129 | 133.0 ± 11.9 | 77.8 ± 10.6 | 119 | 131.2 ± 14.1 | 74.4 ± 11.6 |
| Week 2 | 162 | 125.9 ± 12.7 | 73.8 ± 10.2 | 49 | 128.4 ± 12.4 | 75.8 ± 8.3 | 113 | 124.8 ± 12.8 | 72.9 ± 10.9 |
| Change from baseline  [95% CI] | 160 | −6.4 ± 7.7***  [−7.6, −5.2] | −1.9 ± 4.6***  [−2.7, −1.2] | 49 | −6.8 ± 8.4***  [−9.2, −4.4] | −2.7 ± 5.4***  [−4.3, −1.2] | 111 | −6.2 ± 7.5***  [−7.6, −4.8] | −1.6 ± 4.2***  [−2.4, −0.8] |
| Week 4 | 238 | 124.4 ± 13.2 | 73.4 ± 10.5 | 126 | 126.0 ± 12.2 | 75.1 ± 9.5 | 112 | 122.5 ± 14.0 | 71.5 ± 11.2 |
| Change from baseline  [95% CI] | 234 | −7.5 ± 8.4***  [−8.6, −6.4] | −2.8 ± 5.4***  [−3.5, −2.1] | 123 | −6.9 ± 8.8***  [−8.5, −5.3] | −2.8 ± 6.2***  [−4.0, −1.7] | 111 | −8.2 ± 7.9***  [−9.7, −6.7] | −2.8 ± 4.4***  [−3.6, −2.0] |
| Week 8 | 214 | 121.8 ± 13.2 | 71.6 ± 10.7 | 110 | 123.8 ± 12.4 | 73.5 ± 10.1 | 104 | 119.8 ± 13.7 | 69.5 ± 11.1 |
| Change from baseline  [95% CI] | 211 | −9.9 ± 9.8***  [−11.2, −8.6] | −4.3 ± 6.0***  [−5.1, −3.5] | 108 | −9.3 ± 10.5***  [−11.3, −7.3] | −4.0 ± 6.7***  [−5.3, −2.7] | 103 | −10.5 ± 9.1***  [−12.3, −8.8] | −4.6 ± 5.3***  [−5.7, −3.6] |
| Week 12 | 219 | 121.1 ± 12.4 | 71.3 ± 10.2 | 116 | 122.8 ± 11.6 | 73.1 ± 9.3 | 103 | 119.2 ± 13.1 | 69.2 ± 10.8 |
| Change from baseline  [95% CI] | 215 | −10.8 ± 9.9***  [−12.2, −9.5] | −5.1 ± 6.7***  [−6.0, −4.2] | 113 | −10.6 ± 10.0***  [−12.5, −8.7] | −5.2 ± 7.2***  [−6.6, −3.9] | 102 | −11.1 ± 9.8***  [−13.0, −9.2] | −5.0 ± 6.0***  [−6.2, −3.8] |
| Office BP, mmHg |  |  |  |  |  |  |  |  |  |
| Baseline | 253 | 140.1 ± 13.9 | 78.4 ± 10.3 | 132 | 137.6 ± 14.3 | 79.0 ± 9.8 | 121 | 143.0 ± 12.9 | 77.8 ± 10.9 |
| Week 2 | 105 | 135.3 ± 15.3 | 73.1 ± 9.7 | 28 | 133.9 ± 10.5 | 75.5 ± 8.5 | 77 | 135.8 ± 16.8 | 72.2 ± 10.1 |
| Change from baseline  [95% CI] | 105 | −9.2 ± 12.8***  [−11.7, −6.7] | −3.5 ± 7.9***  [−5.0, −1.9] | 28 | −9.1 ± 11.5***  [−13.6, −4.7] | −1.9 ± 6.3  [−4.3, 0.5] | 77 | −9.2 ± 13.4***  [−12.3, −6.2] | −4.0 ± 8.4***  [−5.9, −2.1] |
| Week 4 | 239 | 131.5 ± 15.3 | 74.9 ± 10.5 | 126 | 130.0 ± 14.6 | 75.9 ± 10.3 | 113 | 133.1 ± 16.0 | 73.7 ± 10.5 |
| Change from baseline  [95% CI] | 239 | −8.3 ± 14.0***  [−10.1, −6.5] | −3.5 ± 9.0***  [−4.7, −2.4] | 126 | −7.1 ± 13.8***  [−9.5, −4.7] | −3.2 ± 8.7***  [−4.7, −1.6] | 113 | −9.7 ± 14.2***  [−12.3, −7.0] | −4.0 ± 9.3***  [−5.7, −2.2] |
| Week 8 | 217 | 130.3 ± 14.5 | 73.3 ± 10.4 | 113 | 128.8 ± 13.8 | 74.0 ± 10.0 | 104 | 131.9 ± 15.1 | 72.7 ± 10.9 |
| Change from baseline  [95% CI] | 217 | −9.4 ± 13.5***  [−11.2, −7.6] | −4.7 ± 8.6***  [−5.9, −3.6] | 113 | −8.2 ± 11.9***  [−10.4, −6.0] | −4.7 ± 8.3***  [−6.3, −3.2] | 104 | −10.8 ± 15.1***  [−13.7, −7.8] | −4.8 ± 8.9***  [−6.5, −3.1] |
| Week 12 | 225 | 128.4 ± 14.1 | 72.8 ± 9.9 | 120 | 127.6 ± 13.3 | 73.2 ± 8.9 | 105 | 129.3 ± 15.0 | 72.4 ± 11.0 |
| Change from baseline  [95% CI] | 225 | −10.9 ± 14.2***  [−12.7, −9.0] | −5.5 ± 9.2***  [−6.8, −4.3] | 120 | −8.9 ± 12.7***  [−11.2, −6.6] | −5.9 ± 8.7***  [−7.4, −4.3] | 105 | −13.0 ± 15.5***  [−16.0, −10.0] | −5.2 ± 9.8***  [−7.1, −3.3] |

Data are mean ± standard deviation.

****P* <0.001 versus baseline, paired *t*-test.

Abbreviations: BP, blood pressure; CI, confidence interval; DBP, diastolic blood pressure; SBP, systolic blood pressure; SGLT2i, sodium–glucose cotransporter-2 inhibitor

**Supplementary Table 5.** Achievement rate (%) of target BP levels at Week 12 (full analysis set)

|  | **Overall**  **N = 243** | **SGLT2i**  **n = 129** | **Non-SGLT2i**  **n = 114** |
| --- | --- | --- | --- |
| Target BP levels: Home SBP/DBP <135/85 mmHg; Office SBP/DBP <140/90 mmHg | | | |
| Morning home SBP/DBP | 71.2 [65.1, 76.8] | 70.5 [61.9, 78.2] | 71.9 [62.7, 79.9] |
| Bedtime home SBP/DBP | 81.5 [76.0, 86.2] | 82.2 [74.5, 88.3] | 80.7 [72.3, 87.5] |
| Office SBP/DBP^1^ | 77.5 [71.8, 82.5] | 83.5 [76.0, 89.3] | 70.7 [61.5, 78.8] |
| Target BP levels: Home SBP <135 mmHg; Office SBP <140 mmHg | | | |
| Morning home SBP | 79.8 [74.2, 84.7] | 79.1 [71.0, 85.7] | 80.7 [72.3, 87.5] |
| Bedtime home SBP | 87.2 [82.4, 91.2] | 87.6 [80.6, 92.7] | 86.8 [79.2, 92.4] |
| Office SBP^1^ | 78.7 [73.1, 83.6] | 83.5 [76.0, 89.3] | 73.3 [64.3, 81.1] |
| Target BP levels: Home DBP <85 mmHg; Office DBP <90 mmHg | | | |
| Morning home DBP | 81.5 [76.0, 86.2] | 80.6 [72.7, 87.0] | 82.5 [74.2, 88.9] |
| Bedtime home DBP | 90.1 [85.7, 93.6] | 90.7 [84.3, 95.1] | 89.5 [82.3, 94.4] |
| Office DBP^1^ | 94.8 [91.2, 97.2] | 96.2 [91.4, 98.8] | 93.1 [86.9, 97.0] |
| Target BP levels: Home SBP/DBP <125/75 mmHg; Office SBP/DBP <130/80 mmHg | | | |
| Morning home SBP/DBP | 31.7 [25.9, 37.9] | 31.0 [23.2, 39.7] | 32.5 [24.0, 41.9] |
| Bedtime home SBP/DBP | 49.0 [42.5, 55.4] | 41.9 [33.2, 50.9] | 57.0 [47.4, 66.3] |
| Office SBP/DBP^1^ | 51.4 [45.0, 57.8] | 57.9 [49.0, 66.4] | 44.0 [34.8, 53.5] |
| Target BP levels: Home SBP <125 mmHg; Office SBP <130 mmHg | | | |
| Morning home SBP | 51.0 [44.6, 57.5] | 51.2 [42.2, 60.1] | 50.9 [41.3, 60.4] |
| Bedtime home SBP | 63.8 [57.4, 69.8] | 58.9 [49.9, 67.5] | 69.3 [60.0, 77.6] |
| Office SBP^1^ | 58.2 [51.8, 64.4] | 63.2 [54.4, 71.4] | 52.6 [43.1, 61.9] |
| Target BP levels: Home DBP <75 mmHg; Office DBP <80 mmHg | | | |
| Morning home DBP | 44.4 [38.1, 50.9] | 38.0 [29.6, 46.9] | 51.8 [42.2, 61.2] |
| Bedtime home DBP | 60.9 [54.5, 67.1] | 52.7 [43.7, 61.6] | 70.2 [60.9, 78.4] |
| Office DBP^1^ | 75.5 [69.7, 80.7] | 78.2 [70.2, 84.9] | 72.4 [63.3, 80.3] |

Data are achievement rates (%) [95% confidence intervals].

^1^ Achievement rate (%) of target office BP levels were calculated using n = 249 for overall, n = 133 for SGLT2i subgroup, and n = 116 for non-SGLT2i subgroup, respectively.

Abbreviations: BP, blood pressure; DBP, diastolic blood pressure; SBP, systolic blood pressure; SGLT2i, sodium–glucose cotransporter-2 inhibitor

**Supplemental Table 6.** Achievement rate (%) of target BP levels at Week 12 (per protocol set)

|  | **Overall**  **N = 219** | **SGLT2i**  **n = 116** | **Non-SGLT2i**  **n = 103** |
| --- | --- | --- | --- |
| Target BP levels: Home SBP/DBP <135/85 mmHg; Office SBP/DBP <140/90 mmHg | | | |
| Morning home SBP/DBP | 70.8 [64.3, 76.7] | 69.0 [59.7, 77.2] | 72.8 [63.2, 81.1] |
| Bedtime home SBP/DBP | 82.6 [77.0, 87.4] | 81.9 [73.7, 88.4] | 83.5 [74.9, 90.1] |
| Office SBP/DBP^1^ | 76.9 [70.8, 82.2] | 81.7 [73.6, 88.1] | 71.4 [61.8, 79.8] |
| Target BP levels: Home SBP <135 mmHg; Office SBP <140 mmHg | | | |
| Morning home SBP | 79.5 [73.5, 84.6] | 77.6 [68.9, 84.8] | 81.6 [72.7, 88.5] |
| Bedtime home SBP | 88.1 [83.1, 92.1] | 87.1 [79.6, 92.6] | 89.3 [81.7, 94.5] |
| Office SBP^1^ | 77.8 [71.8, 83.0] | 81.7 [73.6, 88.1] | 73.3 [63.8, 81.5] |
| Target BP levels: Home DBP <85 mmHg; Office DBP <90 mmHg | | | |
| Morning home DBP | 81.3 [75.5, 86.2] | 79.3 [70.8, 86.3] | 83.5 [74.9, 90.1] |
| Bedtime home DBP | 91.3 [86.8, 94.7] | 91.4 [84.7, 95.8] | 91.3 [84.1, 95.9] |
| Office DBP^1^ | 95.1 [91.4, 97.5] | 95.8 [90.5, 98.6] | 94.3 [88.0, 97.9] |
| Target BP levels: Home SBP/DBP <125/75 mmHg; Office SBP/DBP <130/80 mmHg | | | |
| Morning home SBP/DBP | 33.8 [27.6, 40.5] | 32.8 [24.3, 42.1] | 35.0 [25.8, 45.0] |
| Bedtime home SBP/DBP | 50.2 [43.4, 57.0] | 42.2 [33.1, 51.8] | 59.2 [49.1, 68.8] |
| Office SBP/DBP^1^ | 50.2 [43.5, 56.9] | 56.7 [47.3, 65.7] | 42.9 [33.2, 52.9] |
| Target BP levels: Home SBP <125 mmHg; Office SBP <130 mmHg | | | |
| Morning home SBP | 51.1 [44.3, 57.9] | 50.9 [41.4, 60.3] | 51.5 [41.4, 61.4] |
| Bedtime home SBP | 64.8 [58.1, 71.2] | 59.5 [50.0, 68.5] | 70.9 [61.1, 79.4] |
| Office SBP^1^ | 56.9 [50.1, 63.5] | 61.7 [52.4, 70.4] | 51.4 [41.5, 61.3] |
| Target BP levels: Home DBP <75 mmHg; Office DBP <80 mmHg | | | |
| Morning home DBP | 47.0 [40.3, 53.9] | 40.5 [31.5, 50.0] | 54.4 [44.3, 64.2] |
| Bedtime home DBP | 61.6 [54.9, 68.1] | 52.6 [43.1, 61.9] | 71.8 [62.1, 80.3] |
| Office DBP^1^ | 76.0 [69.9, 81.4] | 79.2 [70.8, 86.0] | 72.4 [62.8, 80.7] |

Data are achievement rates (%) [95% confidence intervals].

^1^ Achievement rate (%) of target office BP levels were calculated using n = 225 for overall, n = 120 for SGLT2i subgroup, and n = 105 for non-SGLT2i subgroup, respectively.

Abbreviations: BP, blood pressure; DBP, diastolic blood pressure; SBP, systolic blood pressure; SGLT2i, sodium–glucose cotransporter-2 inhibitor

**Supplementary Table 7.** Changes and percentage changes (difference between pre-dose values and measurements at Week 12 in the dosing period) on UACR and NT−proBNP in the FAS and PPS

|  | **Overall** | | **SGLT2i** | | **Non-SGLT2i** | |
| --- | --- | --- | --- | --- | --- | --- |
| **Full analysis set** |  | |  | |  | |
| **UACR, mg/gCr** | **n** |  | **n** |  | **n** |  |
| Baseline | 278 | 144.1 ± 413.4 | 145 | 174.1 ± 542.3 | 133 | 111.3 ± 189.1 |
| Week 12 | 247 | 80.0 ± 259.4 | 131 | 100.1 ± 339.3 | 116 | 57.3 ± 113.6 |
| Change from baseline | 246 | −71.5 ± 210.8 | 131 | −86.0 ± 258.9 | 115 | −54.9 ± 136.0 |
| Percentage change in geometric mean from baseline [95% CI] |  | −42.8***  [−48.4, −36.7] |  | −43.0***  [−50.2, −34.8] |  | −42.6***  [−51.0, −32.8] |
| **NT−proBNP, pg/mL** |  |  |  |  |  |  |
| Baseline | 267 | 102.9 ± 156.8 | 140 | 87.4 ± 117.1 | 127 | 119.9 ± 190.4 |
| Week 12 | 238 | 109.1 ± 227.1 | 128 | 94.6 ± 162.1 | 110 | 125.9 ± 284.6 |
| Change from baseline | 229 | 1.5 ± 156.1 | 123 | −3.1 ± 66.7 | 106 | 6.9 ± 218.4 |
| Percentage change in geometric mean from baseline [95% CI] |  | −11.4***  [−16.9, −5.6] |  | −11.3**  [−18.6, −3.5] |  | −11.5*  [−19.7, −2.5] |
| **Per protocol set** |  |  |  |  |  |  |
| **UACR, mg/gCr** | **n** |  | **n** |  | **n** |  |
| Baseline | 252 | 147.9 ± 432.0 | 132 | 183.4 ± 567.6 | 120 | 108.9 ± 189.8 |
| Week 12 | 223 | 83.7 ± 272.3 | 118 | 105.8 ± 356.6 | 105 | 58.8 ± 118.5 |
| Change from baseline | 222 | −71.7 ± 216.6 | 118 | −89.1 ± 271.6 | 104 | −51.9 ± 127.2 |
| Percentage change in geometric mean from baseline [95% CI] |  | −40.5***  [−46.1, −34.3] |  | −40.2***  [−47.9, −31.4] |  | −40.8***  [−48.9, −31.5] |
| **NT−proBNP, pg/mL** |  |  |  |  |  |  |
| Baseline | 243 | 100.2 ± 152.0 | 127 | 90.1 ± 121.5 | 116 | 111.2 ± 179.5 |
| Week 12 | 217 | 102.2 ± 192.1 | 116 | 98.9 ± 169.1 | 101 | 105.9 ± 216.3 |
| Change from baseline | 208 | −3.4 ± 129.6 | 111 | −1.3 ± 69.4 | 97 | −5.8 ± 175.1 |
| Percentage change in geometric mean from baseline [95% CI] |  | −11.4***  [−17.1, −5.3] |  | −9.5*  [−17.2, −1.0] |  | −13.5**  [−21.8, −4.3] |

Data are geometric mean ± standard deviation.

**P* <0.05, ***P* <0.01, ****P* <0.001 versus baseline, paired *t*-test.

For UACR, *p-*values are only presented for percentage change from baseline.

Abbreviations: CI, confidence interval; FAS, full analysis set; NT-proBNP, N-terminal prohormone of brain natriuretic peptide; PPS, per-protocol set; SGLT2i, sodium–glucose cotransporter-2 inhibitor; UACR, urine albumin-to-creatinine ratio

**Supplementary Table 8.** Exploratory endpoints: improvement, ≥30% reduction, and remission rates for UACR (full analysis set)

|  | | **Overall (N = 246)** | | | | | |
| --- | --- | --- | --- | --- | --- | --- | --- |
|  |  | **Sub-cohort at baseline** | | | | | |
|  |  | A1 | A2 | A3 | A1 + A2 | A2 + A3 | A1 + A2 + A3 |
|  |  | n = 136 | n = 76 | n = 34 | n = 212 | n = 110 | n = 246 |
| Week 12 | A1 | － | 29 (38.2) | 2 (5.9) | － | － | － |
|  | A2 | 6 (4.4) | － | 16 (47.1) | － | － | － |
|  | A3 | 0 (0.0) | 0 (0.0) | － | － | － | － |
|  | Improved | － | 29 (38.2) | 18 (52.9) | － | 47 (42.7) | 47 (19.1) |
|  | Worsened | 6 (4.4) | 0 (0.0) | － | 6 (2.8) | － | － |
|  | ≥30% reduction | 71 (52.2) | 52 (68.4) | 26 (76.5) | 123 (58.0) | 78 (70.9) | 149 (60.6) |
|  | Remission^1^ | － | 29 (38.2) | 2 (5.9) | － | 31 (28.2) | － |
|  | | **SGLT2i (n = 131)** | | | | | |
|  |  | **Sub-cohort at baseline** | | | | | |
|  |  | A1 | A2 | A3 | A1 + A2 | A2 + A3 | A1 + A2 + A3 |
|  |  | n = 70 | n = 43 | n = 18 | n = 113 | n = 61 | n = 131 |
| Week 12 | A1 | － | 17 (39.5) | 1 (5.6) | － | － | － |
|  | A2 | 2 (2.9) | － | 8 (44.4) | － | － | － |
|  | A3 | 0 (0.0) | 0 (0.0) | － | － | － | － |
|  | Improved | － | 17 (39.5) | 9 (50.0) | － | 26 (42.6) | 26 (19.8) |
|  | Worsened | 2 (2.9) | 0 (0.0) | － | 2 (1.8) | － | － |
|  | ≥30% reduction | 40 (57.1) | 30 (69.8) | 15 (83.3) | 70 (61.9) | 45 (73.8) | 85 (64.9) |
|  | Remission^1^ | － | 17 (39.5) | 1 (5.6) | － | 18 (29.5) | － |
|  | | **Non-SGLT2i (n = 115)** | | | | | |
|  |  | **Sub-cohort at baseline** | | | | | |
|  |  | A1 | A2 | A3 | A1 + A2 | A2 + A3 | A1 + A2 + A3 |
|  |  | n = 66 | n = 33 | n = 16 | n = 99 | n = 49 | n = 115 |
| Week 12 | A1 | － | 12 (36.4) | 1 (6.3) | － | － | － |
|  | A2 | 4 (6.1) | － | 8 (50.0) | － | － | － |
|  | A3 | 0 (0.0) | 0 (0.0) | － | － | － | － |
|  | Improved | － | 12 (36.4) | 9 (56.3) | － | 21 (42.9) | 21 (18.3) |
|  | Worsened | 4 (6.1) | 0 (0.0) | － | 4 (4.0) | － | － |
|  | ≥30% reduction | 31 (47.0) | 22 (66.7) | 11 (68.8) | 53 (53.5) | 33 (67.3) | 64 (55.7) |
|  | Remission^1^ | － | 12 (36.4) | 1 (6.3) | － | 13 (26.5) | － |

Data are n (%).

A1, UACR <30 mg/gCr

A2, UACR 30–<300 mg/gCr

A3, UACR ≥300 mg/gCr

^1^Defined as transition to A1

Abbreviations: SGLT2i, sodium–glucose cotransporter-2 inhibitor; UACR, urine albumin-to-creatinine ratio

**Supplementary Table 9.** Exploratory endpoints: improvement, ≥30% reduction, and remission rates for UACR (per protocol set)

|  | | **Overall (N = 222)** | | | | | |
| --- | --- | --- | --- | --- | --- | --- | --- |
|  |  | **Sub-cohort at baseline** | | | | | |
|  |  | A1 | A2 | A3 | A1 + A2 | A2 + A3 | A1 + A2 + A3 |
|  |  | n = 133 | n = 57 | n = 32 | n = 190 | n = 89 | n = 222 |
| Week 12 | A1 | － | 21 (36.8) | 0 (0.0) | － | － | － |
|  | A2 | 6 (4.5) | － | 16 (50.0) | － | － | － |
|  | A3 | 0 (0.0) | 0 (0.0) | － | － | － | － |
|  | Improved | － | 21 (36.8) | 16 (50.0) | － | 37 (41.6) | 37 (16.7) |
|  | Worsened | 6 (4.5) | 0 (0.0) | － | 6 (3.2) | － | － |
|  | ≥30% reduction | 70 (52.6) | 38 (66.7) | 24 (75.0) | 108 (56.8) | 62 (69.7) | 132 (59.5) |
|  | Remission^1^ | － | 21 (36.8) | 0 (0.0) | － | 21 (23.6) | － |
|  | | **SGLT2i (n = 118)** | | | | | |
|  |  | **Sub-cohort at baseline** | | | | | |
|  |  | A1 | A2 | A3 | A1 + A2 | A2 + A3 | A1 + A2 + A3 |
|  |  | n = 69 | n = 32 | n = 17 | n = 101 | n = 49 | n = 118 |
| Week 12 | A1 | － | 11 (34.4) | 0 (0.0) | － | － | － |
|  | A2 | 2 (2.9) | － | 8 (47.1) | － | － | － |
|  | A3 | 0 (0.0) | 0 (0.0) | － | － | － | － |
|  | Improved | － | 11 (34.4) | 8 (47.1) | － | 19 (38.8) | 19 (16.1) |
|  | Worsened | 2 (2.9) | 0 (0.0) | － | 2 (2.0) | － | － |
|  | ≥30% reduction | 39 (56.5) | 20 (62.5) | 14 (82.4) | 59 (58.4) | 34 (69.4) | 73 (61.9) |
|  | Remission^1^ | － | 11 (34.4) | 0 (0.0) | － | 11 (22.4) | － |
|  | | **Non-SGLT2i (n = 104)** | | | | | |
|  |  | **Sub-cohort at baseline** | | | | | |
|  |  | A1 | A2 | A3 | A1 + A2 | A2 + A3 | A1 + A2 + A3 |
|  |  | n = 64 | n = 25 | n = 15 | n = 89 | n = 40 | n = 104 |
| Week 12 | A1 | － | 10 (40.0) | 0 (0.0) | － | － | － |
|  | A2 | 4 (6.3) | － | 8 (53.3) | － | － | － |
|  | A3 | 0 (0.0) | 0 (0.0) | － | － | － | － |
|  | Improved | － | 10 (40.0) | 8 (53.3) | － | 18 (45.0) | 18 (17.3) |
|  | Worsened | 4 (6.3) | 0 (0.0) | － | 4 (4.5) | － | － |
|  | ≥30% reduction | 31 (48.4) | 18 (72.0) | 10 (66.7) | 49 (55.1) | 28 (70.0) | 59 (56.7) |
|  | Remission^1^ | － | 10 (40.0) | 0 (0.0) | － | 10 (25.0) | － |

Data are n (%).

A1, UACR <30 mg/gCr

A2, UACR 30–<300 mg/gCr

A3, UACR ≥300 mg/gCr

^1^Defined as transition to A1

Abbreviations: SGLT2i, sodium–glucose cotransporter-2 inhibitor; UACR, urine albumin-to-creatinine ratio

**Supplementary Table 10.** Change in serum potassium and eGFR_creat_ from baseline to Week 12 (safety analysis set)

|  | **Overall** | | **SGLT2i** | | **Non-SGLT2i** | |
| --- | --- | --- | --- | --- | --- | --- |
|  | **n** | **Mean ± SD** | **n** | **Mean ± SD** | **n** | **Mean ± SD** |
| **Serum potassium, mEq/L** |  |  |  |  |  |  |
| Baseline | 282 | 4.2 ± 0.4 | 148 | 4.3 ± 0.4 | 134 | 4.2 ± 0.4 |
| Week 2 | 273 | 4.5 ± 0.4 | 144 | 4.5 ± 0.4 | 129 | 4.4 ± 0.4 |
| Change from baseline | 272 | 0.2 ± 0.3 | 144 | 0.2 ± 0.3 | 128 | 0.3 ± 0.3 |
| Week 4 | 265 | 4.4 ± 0.4 | 140 | 4.4 ± 0.4 | 125 | 4.4 ± 0.4 |
| Change from baseline | 264 | 0.2 ± 0.4 | 140 | 0.1 ± 0.4 | 124 | 0.3 ± 0.4 |
| Week 8 | 238 | 4.4 ± 0.4 | 123 | 4.4 ± 0.4 | 115 | 4.5 ± 0.4 |
| Change from baseline | 237 | 0.2 ± 0.4 | 123 | 0.2 ± 0.4 | 114 | 0.3 ± 0.4 |
| Week 12 | 250 | 4.4 ± 0.4 | 133 | 4.4 ± 0.4 | 117 | 4.4 ± 0.4 |
| Change from baseline | 249 | 0.2 ± 0.4 | 133 | 0.1 ± 0.4 | 116 | 0.3 ± 0.4 |
| **eGFR_creat_, mL/min/1.73 m^2^** |  |  |  |  |  |  |
| Baseline | 282 | 61.7 ± 18.0 | 148 | 65.1 ± 19.6 | 134 | 58.0 ± 15.4 |
| Week 2 | 273 | 59.0 ± 18.1 | 144 | 61.4 ± 19.8 | 129 | 56.3 ± 15.6 |
| Change from baseline | 272 | −3.3 ± 7.0 | 144 | −3.9 ± 7.2 | 128 | −2.6 ± 6.7 |
| Week 4 | 265 | 58.2 ± 18.1 | 140 | 60.7 ± 19.6 | 125 | 55.5 ± 15.9 |
| Change from baseline | 264 | −4.0 ± 7.2 | 140 | −4.6 ± 7.6 | 124 | −3.3 ± 6.6 |
| Week 8 | 238 | 56.8 ± 17.5 | 123 | 59.0 ± 18.8 | 115 | 54.4 ± 15.6 |
| Change from baseline | 237 | −4.2 ± 7.0 | 123 | −4.6 ± 6.8 | 114 | −3.8 ± 7.2 |
| Week 12 | 250 | 57.7 ± 17.8 | 133 | 60.8 ± 19.2 | 117 | 54.3 ± 15.5 |
| Change from baseline | 249 | −4.7 ± 7.6 | 133 | −4.9 ± 8.1 | 116 | −4.5 ± 7.0 |

*P*-values were not calculated.

Abbreviations: eGFR_creat_, estimated glomerular filtration rate (creatinine-based); SD*,* standard deviation; SGLT2i, sodium–glucose cotransporter-2 inhibitor

**Supplementary Table 11.** Incidence of serum potassium level ≥5.5 mEq/L (safety analysis set)

|  | **Overall**  **N = 283** | **SGLT2i**  **n = 148** | **Non-SGLT2i**  **n = 135** |
| --- | --- | --- | --- |
| Serum potassium ≥5.5 mEq/L, n (%) | 10 (3.5) | 3 (2.0) | 7 (5.2) |

Abbreviation: SGLT2i, sodium–glucose cotransporter-2 inhibitor
